# Pharmacologic inhibition of dipeptidyl peptidase 1 (cathepsin C) does not block *in vitro* granzyme-mediated target cell killing by CD8 T or NK cells

**DOI:** 10.3389/fphar.2024.1396710

**Published:** 2024-07-03

**Authors:** Vivien R. Sutton, Sally V. Watt, Hedieh Akhlaghi, David C. Cipolla, Kuan-Ju Chen, Daniel LaSala, Patrick P. McDonald, Paul A. Beavis, Isabelle Munoz, Adrian W. Hodel, Tahereh Noori, Ilia Voskoboinik, Joseph A. Trapani

**Affiliations:** ^1^ Cancer Immunology Program, Peter MacCallum Cancer Centre, Melbourne, Australia; ^2^ Sir Peter MacCallum Department of Oncology, The University of Melbourne, Melbourne, Australia; ^3^ Insmed Incorporated, Bridgewater, NJ, United States

**Keywords:** DPP1, granzyme, apoptosis, serine protease, T cell, lysosome, protease inhibitor, granule exocytosis

## Abstract

Recently developed small-molecule inhibitors of the lysosomal protease dipeptidyl peptidase 1 (DPP1), also known as cathepsin C (CatC), can suppress suppurative inflammation *in vivo* by blocking the processing of zymogenic (pro-) forms of neutrophil serine proteases (NSPs), including neutrophil elastase, proteinase 3, and cathepsin G. DPP1 also plays an important role in activating granzyme serine proteases that are expressed by cytotoxic T lymphocytes (CTL) and natural killer (NK) cells. Therefore, it is critical to determine whether DPP1 inhibition can also cause off-target suppression of CTL/NK-cell-mediated killing of virus-infected or malignant cells. Herein, we demonstrate that the processing of human granzymes A and B, transitioning from zymogen to active proteases, is not solely dependent on DPP1. Thus, the killing of target cells by primary human CD8^+^ T cells, NK cells, and gene-engineered anti-CD19 CAR T cells was not blocked *in vitro* even after prior exposure to high concentrations of the reversible DPP1 inhibitor brensocatib. Consistent with this observation, the turnover of model granzyme A/B peptide substrates in the human CTL/NK cell lysates was not significantly reduced by brensocatib. In contrast, preincubation with brensocatib almost entirely abolished (>90%) both the cytotoxic activity of mouse CD8^+^ T cells and granzyme substrate turnover. Overall, our finding that the effects of DPP1 inhibition on human cytotoxic lymphocytes are attenuated in comparison to those of mice indicates that granzyme processing/activation pathways differ between mice and humans. Moreover, the *in vitro* data suggest that human subjects treated with reversible DPP1 inhibitors, such as brensocatib, are unlikely to experience any appreciable deficits in CTL/NK-cell-mediated immunities.

## Introduction

Dipeptidyl peptidase I (DPP1, cathepsin C, CatC) is a cysteine protease that catalyzes the activation of many serine proteases from their zymogenic to active forms through the removal of an amino-terminal activation dipeptide (Thiele and Lipsky, 1990; McGuire et al., 1992; Brown et al., 1993; McGuire et al., 1993). In the immune system, this includes serine proteases expressed specifically in myeloid cells (especially in neutrophils), such as neutrophil elastase (NE), proteinase 3 (PR3), and cathepsin G (CatG), and in the lymphoid cells, they constitute a family of serine proteases known as granzymes. The degranulation of neutrophils at the sites of bacterial infection results in accumulation of high concentrations of NE, PR3, and CatG in the host tissues, where their bactericidal and proinflammatory activities limit bacterial proliferation (Pham, 2006).

As with neutrophil serine proteases, granzymes are released by exocytosis. Here, the lymphoid effector cells are triggered to release granzymes following the formation of stable immune synapses between the cytotoxic (killer) lymphocytes such as CD8^+^ cytotoxic T lymphocytes (CTL) or natural killer (NK) cells and cognate target cells (Voskoboinik et al., 2015). Granzymes released into the immune synapses formed with virus-infected or malignant cells act in synergy with the pore-forming protein toxin perforin to induce target cell apoptosis as well as a range of additional proinflammatory effects (Sutton et al., 2000; Sutton et al., 2003; Lopez et al., 2013). The neutrophil serine proteases and granzymes share close structural and catalytic similarities as they have evolved from more ancient digestive proteases such as chymotrypsin (Smyth et al., 1996; Susanto et al., 2012). Despite their very tight cell-specific expressions, the genes encoding neutrophil and lymphocyte serine proteases are colocated. In humans, the granzyme B (*GZMB*) and CatG (*CATG*) genes are separated by only 30 kilobases on Chr 14, while the genes for NE (ELANE), PR3 (PRTN3), and granzyme M (*GZMM*) are closely linked on Chr 19. This indicates the close evolutionary relationship between the two protease families, which is also reflected in syntenic gene arrangements in species as distant from humans as rats and mice (Smyth et al., 1996).

In addition to their close structural and genetic relationships, the serine proteases of myeloid cells and lymphocytes share very similar mechanisms of activation, intracellular trafficking, storage, and secretion (Bird et al., 2009). Following mRNA translation in the endoplasmic reticulum, a signal peptide of 18–20 residues that is found in all proteins destined for the secretory pathway is removed at the N terminus, resulting in a protease proform. Final processing of the inactive proform requires removal of two residues at the amino terminus by DPP1 or another enzyme, which is necessary for accurate folding of the N terminus into the body of the protease close to the protease active site to achieve full catalytic activity (McGuire et al., 1993; Bird et al., 2009).

Given its indispensable role in activating neutrophil proteases, DPP1 is considered an attractive target for attenuating the inflammatory cycle in patients with chronic infections like bronchiectasis (Korkmaz et al., 2010) or other chronic diseases with inflammatory components like lupus nephritis or rheumatoid arthritis (Korkmaz et al., 2010; Chen et al., 2023; McDonald et al., 2023). In bronchiectasis, lung scarring caused by severe pneumonia or other chronic inflammatory conditions such as cystic fibrosis hinders mucus clearance from large airways, leaving the host susceptible to recurrent bacterial infections (Martins et al., 2023). Mucus may also accumulate in the saccular dilatations of the airways owing to congenital lung malformations or spinal deformities such as kyphoscoliosis that can only be cleared by intensive physiotherapy, chest percussion, and postural drainage. High levels of NE in the lung sputum are associated with greater morbidity and mortality in bronchiectasis (Chalmers et al., 2017; Cipolla et al., 2023). Thus, DPP1 inhibition is expected to reduce lung suppuration in these conditions and reduce acute infective exacerbations that require antibiotics and/or inpatient management. Indeed, the reversible small-molecule DPP1 inhibitor brensocatib attenuates NE, PR3, and CatG activities in the sputum in a dose-dependent manner and prolongs the time to first exacerbation of the disease, as noted in a phase II clinical trial (WILLOW; NCT0321891) in non-cystic fibrosis bronchiectasis patients (Chalmers et al., 2020, 2022; Cipolla et al., 2023). Brensocatib is currently being investigated in a multicenter phase III clinical trial (ASPEN; NCT04594369) to evaluate its capacity to reduce the frequency and/or severity of acute infective episodes (i.e., exacerbations) in the lungs of patients with bronchiectasis.

Given that DPP1 also plays a significant role in progranzyme processing/activation, the purpose of the current study was to determine the impact of brensocatib on CTL/NK cell functions and to explore the potential for any undesirable effects of DPP1 inhibition on cytotoxic lymphocyte functions. The undesirable effects of long-term DPP1 inhibition theoretically include vulnerability to certain viral infections against which granzymes play protective roles as well as survival of transformed cells that are normally deleted by immunotherapies that activate anti-cancer CTLs (Voskoboinik et al., 2015). These include administration of checkpoint blockade antibodies or adoptive transfer of gene-engineered anti-cancer chimeric antigen receptor (CAR) T cells (Trapani and Darcy, 2017). As expected, the neutrophils and monocytes of mice devoid of DPP1 expression owing to targeted disruption of both of their CatC alleles (CatC−/−) completely lacked CatG activities (Pham and Ley, 1999; Sutton et al., 2007). The killing of cancer cells by CD8^+^ T cells and NK cells purified from the same mice was also markedly reduced, and the reduced granzyme activity was reflected in the slower turnover of model peptide substrates specific to granzyme A (GzmA) and granzyme B (GzmB). Reductions in the granzyme activities were greater for GzmA than GzmB (Pham and Ley, 1999; Sutton et al., 2007).

The extent to which CTL/NK-cell-mediated target cell death depends on DPP1-mediated processing of granzymes has not been reported experimentally in human subjects. However, some insights into this issue may be derived from studying patients with congenital DPP1 deficiency (Pham et al., 2004; Pham, 2006). In the autosomal recessive immunodeficiency Papillon–Lefevre syndrome (PLS), the affected children almost invariably present with intractable suppurative gum infections (chronic gingivitis) that lead to loss of teeth by the age of 6–8 years (Pham et al., 2004; Pham, 2006). DPP1 is also expressed at high levels in the skin, and marked thickening of the skin of the palms and soles (hyperkeratosis) can result from failed keratinocyte desquamation at these sites. Despite these florid clinical features, disorders of cytotoxic lymphocyte functions have not been reported in PLS, even in cases where DPP1 activity is completely abolished (Bird et al., 2009; Susanto et al., 2012). Although this finding suggests that pharmacological DPP1 inhibition should be tolerated well, the present study aimed to formally investigate this issue.

## Materials and methods

### Cell lines

Mouse (P815, EL4, EL4.huCD19, EL4.moCD19, and MC57) and human (K562, Jurkat, HepG2, and MINO) cells were maintained in DME and RPMI media, respectively, and were supplemented with 2 mM L-GlutaMAX™ (Gibco, United States), 50 mg/mL penicillin, 50 mg/mL streptomycin, 50 mM 2-mercaptoethanol, and 10% (v/v) fetal bovine serum (FBS).

### Preparation of mouse cytotoxic T lymphocytes

H-2K^b^-restricted T-cell receptor transgenic mice directed against the SIINFEKL peptide of ovalbumin (C57BL/6.OTI, OTI) were bred and maintained at the Peter MacCallum Cancer Centre, Melbourne, Australia. The animal studies were approved through the AEEC ethics application E651. Single-cell suspensions prepared from the spleens of the OTI mice were cultured in RPMI medium additionally supplemented with 100 mM non-essential amino acids, 1 mM sodium pyruvate, 100 U/mL human rIL-2 (NCI, Frederick, MD, United States), and 1 mM SIINFEKL peptide (Hogquist et al., 1994). For the cytotoxicity experiments, the cell cultures were supplemented with either brensocatib (10 µM final concentration) or dimethylsulfoxide (DMSO; diluent) at time = 0. The cells were harvested, washed, and resuspended in fresh culture medium supplemented with fresh drug every 2–3 days until used in the cytotoxicity experiments, typically after 5 days in culture.

### Preparation of primary human NK, LAK, and T cells

All human studies were approved by the Human Research Ethics Committee of Peter MacCallum Cancer Centre (HREC 01/14) and abided by the principles of the Declaration of Helsinki. Human peripheral blood mononuclear cells (PBMCs) were isolated from healthy donor blood samples by separation on Ficoll cushions. To generate the NK cells, the PBMCs were cultured overnight in RPMI medium containing 100 U/mL of human rIL-2; for the lymphokine activated killer (LAK) cells, the RPMI medium contained 1000 U/mL of human rIL-2 cultured for 8 days; for the activated CD4^+^ and CD8^+^ T cells, the RPMI medium contained anti-CD2, anti-CD3, and anti-CD28 antibodies (Immunocult T cell activator kit; Stem cell, #10970) along with 100 U/mL of human rIL-2 cultured for 4 days.

### Generation of human CAR T cells

Human CAR T cells directed against human CD19 were produced by transduction of human T cells with a retroviral supernatant, as described previously (Hagn et al., 2014). Briefly, the PBMCs were stimulated with anti-human CD3 (OKT3 30 ng/mL, Invitrogen # 16-0037–81) and IL-2 (100 U/mL) for 2 days. Following this, the activated PBMCs were induced to express the anti-hCD19 (clone FMC63) scFv_myc_hCD8hCD28_hCD3ζ CAR subcloned into a pSAMEN vector backbone. The culture supernatant of stably expressing PG13 cells was used as the source of the retrovirus. Retronectin (Takara Bio, Kusatsu, Shiga, Japan) was coated on culture plates and utilized in the retroviral transductions according to manufacturer instructions. Briefly, the retroviral supernatant from the PG13 producer cells was placed on the retronectin-coated plates (15 μg/mL) by centrifugation (2000*g*, 1 h), followed by addition of T cells (1–2 ×10^6^ per mL of viral supernatant). The cultures were also supplemented with either brensocatib (10 µM final concentration) or DMSO diluent throughout the culture period. The drug or diluent was also included at the corresponding concentration in all cytotoxicity assays, as detailed below. The transduced CD8^+^ CAR T cells were selected by FACs sorting 3 days later upon staining with an antibody to detect the MYC epitope incorporated in the anti-CD19 CARs.

### Generation of mouse CAR T cells

All animal studies were approved under project E651 by the Animal Ethics Committee of Peter MacCallum Cancer Centre, Melbourne, Australia. Mouse CAR T cells directed against mouse CD19 were obtained by transduction of mouse OTI spleen cells with the retroviral supernatant from pLXSN_mCD19_MYC_CD8mCD28_Zeta GP86+ cells, similar to that described for the human CAR T cells (Westwood et al, 2005). The T cells were activated with 100 U/mL of human rIL-2 (NCI, Frederick, MD, United States) and in some instances, with 1 µM of SIINFEKL peptide. The cultures were supplemented with either brensocatib (10 µM final concentration) or DMSO diluent throughout the culture period. The transduced CD8^+^ CAR T cells were then selected by FACs sorting in a similar manner as that noted before.

### Cytotoxicity assays

The deaths of the target cells in response to human or mouse T, NK, or CAR T cells were measured in 4-h ^51^Cr release assays, as described previously (Rudd-Schmidt et al., 2019). Functional immune synapses were formed between the human cytotoxic T cells and Fcγ-receptor-positive P815 target cells by the addition of IgG anti-huCD3 antibody (1 mg/mL OKT3, Invitrogen # 16-0037–81) (Noori et al., 2022). In this assay, cell death is possible only upon stable conjugation of the T cells and target cells via the intermediary anti-CD3 IgG; omission of the IgG from the assay completely abrogates cell death (Noori et al., 2022). Brensocatib or DMSO diluent was appropriately added to the corresponding coculture.

### Granzyme substrate turnover assays

Postnuclear cell lysates were prepared from the washed CTL and NK cell cultures and normalized for the protein content. Hydrolysis of the synthetic peptide thiobenzylester substrates specific for GzmB [Boc-Ala-Ala-Asp (AAD)-SBzl; SM Biochemicals, Anaheim, CA, United States) or GzmA [Boc-Lys-SBzl (BLT); Sigma-Aldrich] was used to quantify the granzyme protease activity, as reported previously (Davis et al., 2003; Hagn et al., 2014).

### CRISPR Cas9-mediated disruption of cathepsin H (*CATH*) gene in human T cells

The sequences of synthetic guide (sg) RNAs complementary to the opposite DNA strands at the 5′ and 3’ ends of exon 3 of the human *CATH* gene were generated using the Synthego CRISPR Design tool (Supplementary Figure). Human T cells purified from the PBMCs of healthy human donors (EasySep Human T cell Isolation kit, Cat#17951, Stemcell Technologies) were electroporated with the Cas9 protein (Cat#1081059, Integrated DNA Technologies) and sgRNAs using the Lonza 4D-Nucleofector Core Unit (Lonza, Cat# AAF-1002B) and P3 primary cell 4D-Nucleofector electroporation kit (Lonza, Cat# V4XP- 3032), as described previously (Nussing et al., 2020). The cells were recovered at 37°C for 10 min and transferred to 24-well plates containing either 10 µM brensocatib or an equivalent concentration of the diluent (DMSO) in RPMI medium containing activating anti-T-cell antibodies (ImmunoCult™ Human CD3/CD28/CD2 T Cell Activator) along with 100 U/mL of human rIL-2. The control non-electroporated T cells were also cultured with either brensocatib or DMSO.

### Validation of human *CATH* gene disruption

Genomic DNA purified from the CRISPR knockout and control cell cultures using the Qiagen DNAeasy Blood and Tissue kit protocol (Cat no: 69,504) was used as the template in the polymerase chain reaction (PCR) using sense and antisense primers at the opposite ends of exon 3. The PCR products were sequenced by the Australian Genome Research Facility (AGRF Inc., Brisbane) and compared to wild-type gene sequences using the Inference of CRISPR Edits (ICE) tool (Synthego).

### Western blotting

Whole cell lysates (30 µg/lane) were electrophoresed on BOLT 4%–12% Bris-Tris Plus 1.0 mm × 15-well gels (Invitrogen by Thermo Scientific, NW04125BOX, Carlsbad, CA) and transferred to nitrocellulose membranes (IPVH00010, Sigma-Aldrich). Polyclonal rabbit anti-cathepsin H antibody (PA551951, ThermoFisher Scientific, diluted 1/500) was used, and the bound IgG was detected with HRP-conjugated swine anti-rabbit/HRP (1/1000; Dako Cat no: P0217). Chemiluminescence was generated and measured with ECL Western blotting detection reagents (RPN2106, Cytiva, Amersham, Buckinghamshire, UK) on an iBright 1500 imaging system (Invitrogen, Thermo Scientific).

### RT-PCR of human cathepsin H mRNA

RNA from the human *CATH* gene-disrupted T cells was isolated (Zymo Research Direct-zol RNA kit; Integrated Science, Victoria, Australia), and cDNA was generated by reverse transcriptase (Applied Biosystems) according to the manufacturer’s protocols. The PCR products were generated using primers (Integrated DNA Technologies, Singapore) designed using the Snapgene Viewer and were separated in 1% (w/v) agarose gels before being visualized on a Molecular Imager Gel Doc XR + Imaging system (Biorad).

### Human, rat, mouse, and dog DPP1 enzyme IC50 assays

Recombinant human, rat, and mouse DPP1 enzymes were sourced commercially (R&D Systems; Minneapolis, MN). The human and rat enzymes were proteolytically processed into their mature forms using recombinant human cathepsin L (R&D Systems) in a buffer containing 20 mM of citric acid at pH 4.5, 150 mM of NaCl, 1 mM of EDTA, and 10 mM of DTT. Dog DPP1 enzyme was then obtained from white blood cell lysates enriched from canine whole blood (BioIVT; Westbury, NY). Brensocatib was applied to the DPP1 enzymes in the assay buffer (25 mM of MES at pH 6.0 for the human, rat, and dog assays; 50 mM of MES at pH 5.5 for the mouse assay; 50 mM of NaCl and 5 mM of DTT) at DPP1 concentrations of 1 ng/μL for human, 64 pg/μL for rat, and 62.5 pg/μL for mouse. Since the dog DPP1 enzyme was not pure and was part of a crude lysate, the amount of DPP1 enzyme used was based on a substrate turnover comparable to those of the other test species. Brensocatib at various concentrations (0.08–5000 nM) was preincubated with each DPP1 enzyme for 10 min at 37°C, after which the H-Gly-Arg-AMC substrate (Bachem; St. Torrance, CA) was added to obtain a final substrate concentration of 400 µM. Substrate cleavage was then measured for 90 min at 37°C with fluorescence at excitation/emission wavelengths of 350/450 nm measured every 5 min. The DPP1 concentrations were interpolated based on their activities relative to standard curves of the activated DPP1 enzymes. The IC50 values were then calculated via the XLFit (IDBS Version 5.3.1.3) add-on to Microsoft Excel using the following four-parameter fit equation

$$y=(A+(\left(B-A\right)/(1+((C/x{)}^{\wedge }D))))$$

that is available as equation number 205 (4 parameter logistic model or sigmoidal dose-response model) in XLFit. The default constraints were used for each parameter. The IC50 for brensocatib was defined as the concentration at which 50% of the enzyme activity was inhibited when compared to that of the control (see also Palmer et al., 2018).

### Human cathepsin H enzyme IC50 assay

Cathepsin H (CatH) purified from the human liver (Enzo Life Sciences; Farmingdale, NY) was used for compound testing in a buffer containing 200 mM of KH_2_PO_4_, 200 mM of Na_2_HPO_4_ at pH 6.8, 4 mM of EDTA, and 40 mM of cysteine. Brensocatib in the assay buffer along with 5% DMSO was first added to the human CatH enzyme at a concentration of 3 ng/μL and allowed to preincubate for 10 min at 37°C, after which the L-arginine-7-amido-4-methylcoumarin hydrochloride substrate (Millipore Sigma; Burlington, MA) was added to obtain a final concentration of 150 µM and final DMSO concentration of 1%. Substrate cleavage was then measured for 90 min at 37°C using fluorescence at excitation/emission wavelengths of 370/460 nm measured every 5 min. The CatH concentration was interpolated based on its activity relative to a standard curve of the CatH enzyme. The IC50 value for brensocatib against CatH was calculated in the same manner as that for the DPP1 enzyme IC50 assays.

## Results

**FIGURE 1:**
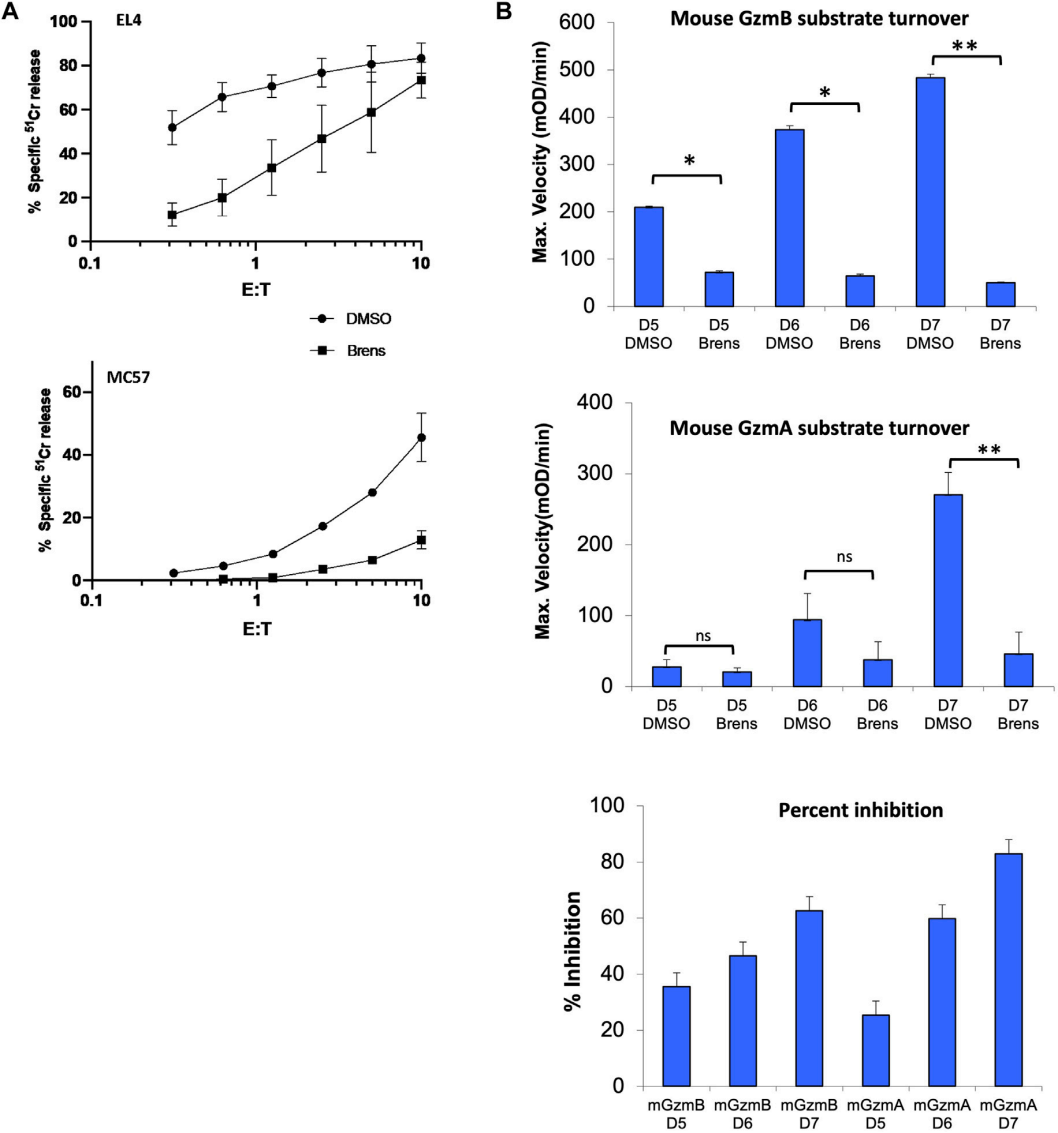
**(A)** Killing of SIINFEKL peptide-pulsed EL4 and MC57 target cells incubated with CD8^+^ splenic T lymphocytes cultured *in vitro* for 5 days. Cytotoxicity was quantified across the range of effector: target (E/T) ratios shown in terms of the percentage of specific release of preloaded cytoplasmic ^51^Cr from the target cells relative to complete lysis with 1.0 M HCl (100%) and spontaneous ^51^Cr release in the absence of CD8^+^ lymphocytes (0%). The values shown are the mean ± SEM across three independent experiments, with each data point in each experiment estimated in triplicate. **(B)** Cleavage of tripeptide substrates specific to GzmB or GzmA in the lysates of splenic OTI T cells stimulated *in vitro* for 5, 6, or 7 days (D5-7). All OTI cell cultures were in the continuous presence of 10 µM brensocatib (Brens) or DMSO diluent. The values are shown as maximum reaction velocity and are the mean of three independent experiments. The mean percentage inhibition of substrate turnover by brensocatib relative to the diluent for each time point in the top two panels is displayed in the bottom panel. *p < 0.05; **p < 0.01 by unpaired *t*-test. ns, not significant.

It was previously shown that the CD8^+^ T lymphocytes from CatC−/− mice have markedly reduced but not completely abolished cytotoxicity for cognate target cells (Pham, 2006; Sutton et al., 2007). To determine whether the pharmacological inhibition of DPP1 would phenocopy this defect in function, lymphocytes freshly isolated from the spleens of wild-type C57BL/6.OTI mice were activated *in vitro* for 5 days in the continual presence of 10 µM brensocatib (or diluent, DMSO); this drug concentration is more than 500-fold that of the IC50 value for purified human DPP1 protease (17.1 nM) and approximately 400-fold that of the IC50 for recombinant mouse DPP1 protease (24.1 nM) (Table 1). The CD8^+^ T cells of these transgenic mice express a single clonogenic T cell receptor that enables them to recognize the SIINFEKL peptide corresponding to amino acids 257–264 of chicken ovalbumin (OVA) presented on the major histocompatibility complex molecule H2-K^b^. No target cell deaths occur in the absence of SIINFEKL (Hogquist et al., 1994; Jenkins et al., 2015). Continuous exposure of the activated T cells to brensocatib for 5 days had no effects on CD8^+^ T cell viability, proliferation, or expressions of activation markers CD69, CD44, and CD62L (data not shown). However, the deaths of the SIINFEKL peptide pulsed EL4 (mouse T cell lymphoma) and MC57 (mouse colorectal cancer) target cells were greatly reduced in comparison to the T cells exposed only to the drug diluent (Figure 1A). By comparing the ratio of CD8^+^ T cells to target cells required to achieve a given level of ^51^Cr release, we estimated that 10 µM of brensocatib inhibited the deaths of SIINFEKL-pulsed EL4 and MC57 cells by approximately 90% (Figure 1A). The cytotoxicity was inhibited to a similar extent irrespective of the CD8^+^ T cells being cultured in a medium containing brensocatib for 4, 5, 6, or 7 days (data not shown).

**TABLE 1:** IC50 values of brensocatib for human, dog, rat, and mouse DPP1 enzymes as well as human CatH enzyme assays^a^.

	*In vitro* IC50				
	DPP1				CatH
	Human	Dog (nM)	Rat (nM)	Mouse (nM)	Human
Brensocatib	17.1 nM	38.1	51.1	24.1	>100 µM

^a^ Recombinant human, rat, and mouse DPP1 enzymes were used to measure brensocatib’s IC50 values. For the dog case, DPP1 was extracted from dog whole blood white blood cell lysates since an appropriate source of recombinant dog DPP1 enzyme was not available. The CatH enzyme was purified from human liver; n > 3.

To determine whether loss of cytotoxicity coincided with the reduced granzyme activity, lysates of the activated CD8^+^ T cells were tested for model short peptide substrate turnover indicating cleavage by GzmA (after Lysine) or GzmB (after Aspartate). GzmB activity in the diluent-treated cells was readily detectable by day 5 and increased progressively by day 7 but was reduced by >80% when the CD8^+^ T cells were cultured with brensocatib (Figure 1B). In a typical manner, GzmA activity was detectable later during the culture period (day 6) and increased further by day 7, but the substrate turnovers at both timepoints were again strongly inhibited by brensocatib (Figure 1B). All in all, the reduced granzyme activity in response to brensocatib was consistent with the inhibition of target cell death observed for the splenic T cells of the *CatC*-gene-disrupted mice.

**FIGURE 2:**
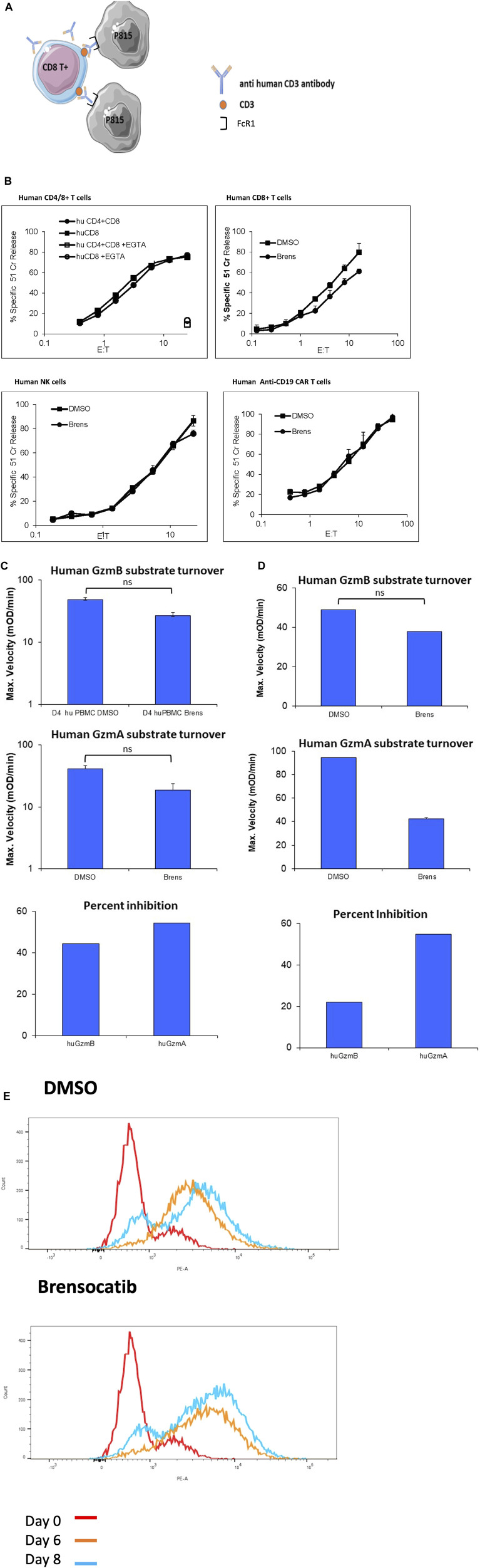
**(A)** Schematic depiction of the redirected P815 cytotoxicity assay. In this assay, functional immune synapses are formed between the ^51^Cr-loaded P815 target cells and activated human CD8^+^ CTLs through the addition of an anti-human CD3 IgG monoclonal antibody that binds CD3 through its antigen-binding VH and VL domains and to the FcgR1 on the P815 cells via the Fc portion. **(B)** Cytotoxicity by human CD3/4/8^+^ activated T cells, IL-2 activated NK cells, and CAR T cells is EGTA-inhibitable but not reduced by brensocatib. In each case, the cytotoxicity is shown for the range of effector: target (E/T) ratios shown, expressed as the percentage of specific release of preloaded cytoplasmic ^51^Cr from the target cells relative to complete lysis with 1.0 M HCl (defined as 100%) and spontaneous ^51^Cr release in the absence of CD8^+^ lymphocytes (defined as 0%). Top left, EGTA was added to cocultures with the highest E/T ratio and completely blocked target cell death. The values shown are the mean ± SEM across three independent experiments, with each data point in each experiment estimated in triplicate. **(C)** Cleavage of tripeptide substrates specific to GzmB or GzmA in the lysates of human CD8^+^ CTLs or **(D)** IL-2-activated human NK cells. All cell cultures were in the continuous presence of 10 µM brensocatib (Brens) or DMSO diluent. The values are shown as maximum reaction velocity and are the mean of three independent experiments. The mean percentage inhibition of substrate turnover by brensocatib in the top two panels is displayed in each bottom panel. **(E)** Intracellular FACS staining for human GzmB in unstimulated human CD8^+^ CTLs (day 0) or in activated T cells exposed to 10 mM brensocatib or DMSO diluent for the number of days indicated. ns, not significant.

We next performed similar experiments using human cytotoxic lymphocytes. To test whether killing by human CD8^+^ CTLs is inhibited by brensocatib exposure, we first activated the peripheral blood T cells of healthy donors by incubating with agonistic antibodies to CD3 (augmenting T cell receptor signaling) and CD28 (costimulatory signaling). To quantify the polyclonal CD8 T cell cytotoxicity, we utilized a well-validated antibody-dependent T cell cytotoxicity assay against ^51^Cr-labeled P815 target cells (Noori et al., 2022). In this assay, functional immune synapses are formed by mixing the CD3^+^ human T cells with FcγR1+ P815 target cells precoated with anti-CD3 antibody; no target cell death occurs in the absence of bridging IgG (Noori et al., 2022) (Figure 2A). The deaths of the P815 cells were shown to be perforin-/granzyme-dependent as complexing the free calcium in the medium with EGTA completely blocked cell death, whether in response to purified CD8^+^ T cells or a mixture of CD4^+^ and CD8^+^ T cells (Figure 2B, top left, shown for the highest E/T ratio of 20:1). Unlike the mouse CD8^+^ T cells, cytotoxicity by human CTLs was minimally diminished by 10 μM brensocatib despite addition of the drug for the entire period of T cell culture (Figure 2B, top right). As a second source of human CTLs, we tested IL-2-activated peripheral blood NK cells that were also continuously cultured in a medium containing brensocatib (LAK cells). We found that the deaths of the K562 target cells were not reduced by brensocatib (Figure 2B, bottom left). Finally, we generated retrovirus-transduced anti-human CD19 CAR T cells from freshly isolated human peripheral blood lymphocytes and again found that the inclusion of brensocatib in the culture medium did not reduce the deaths of the CD19^+^ MINO target cells (Figure 2B, bottom right). The failure of brensocatib to block the cell deaths imparted by the three different types of human CTLs strongly suggests that significant granzyme activity remained. This was confirmed in that the substrate turnovers for both human GzmA and GzmB were reduced but not abrogated in the activated CD8^+^ T CTLs (40%–60%, Figure 2C) and IL-2 activated NK cells (20%–60%, Figure 2D), respectively, compared to the mouse CD8^+^ T cells (reduced >90%, Figure 1).

Of relevance to the clinical studies is the fact that the concentration of brensocatib used in the *in vitro* experiments (10 μM) is more than 30-fold higher than the 0.28 μM maximum plasma concentration (mean Cmax) achieved at steady state in the population pharmacokinetic model of non-cystic-fibrosis bronchiectasis patients receiving brensocatib at the highest dose of 25 mg (Chalmers et al., 2022) being evaluated in the phase III clinical trial (ASPEN; NCT04594369). As noted above, this blood level significantly reduces NE and CatG activities in the sputum of the human participants and delays the recurrence of infective episodes. We also showed that exposure to brensocatib expectedly had no significant impact on the quantity of immunoreactive GzmB stored in the activated T cells (Figure 2E).

**FIGURE 3:**
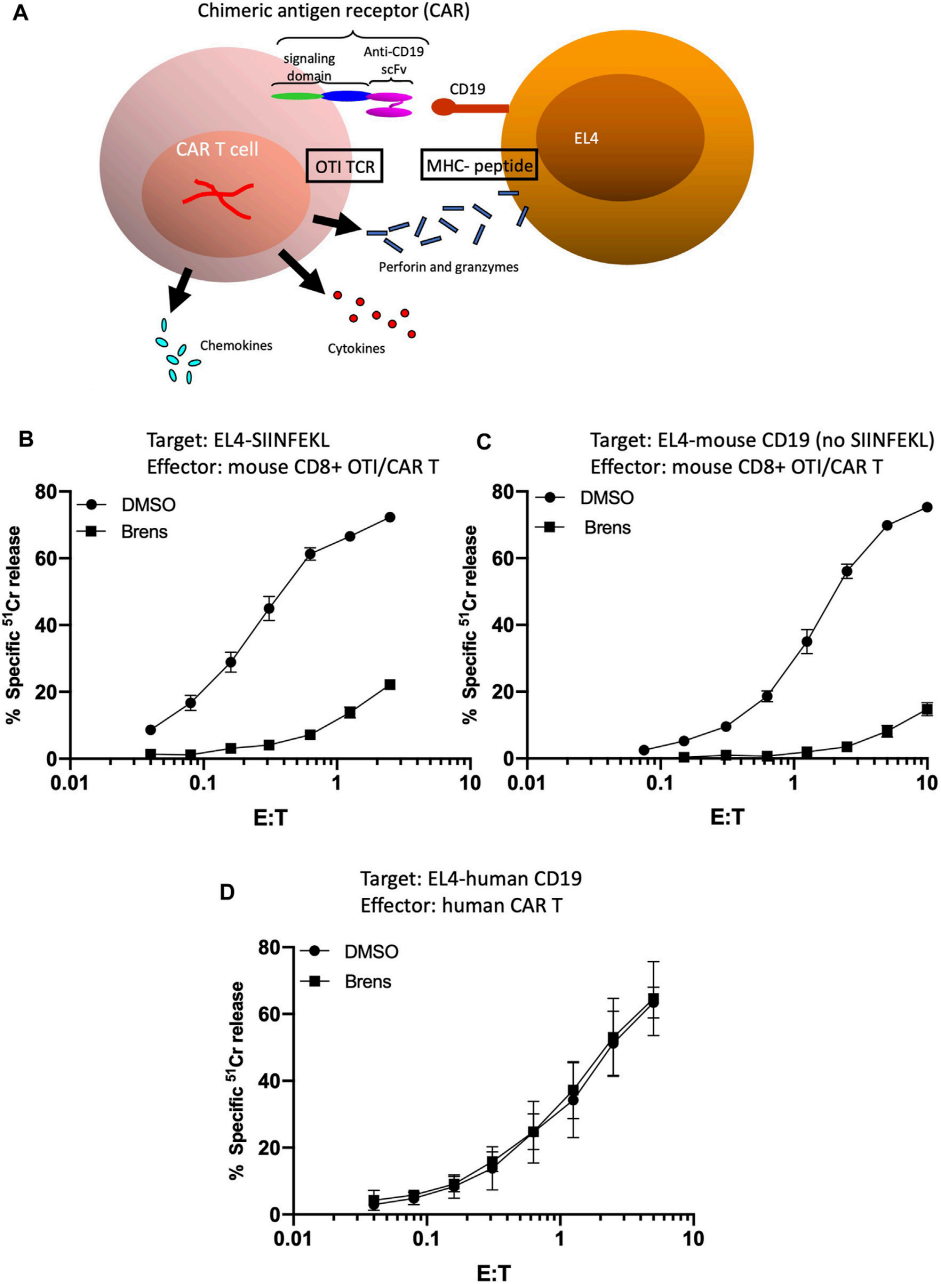
**(A)** Schematic depiction of mouse OTI CTLs engineered to have dual target cell specificity. Variants of EL4 were generated to stably express either mouse or human CD19. Mouse and human CD8^+^ CAR T cells were then generated so as to express CARs with specificity for mouse or human CD19, respectively. By utilizing CD8^+^ T cells from OTI transgenic mice as the starting population for production of mouse CAR T cells, it was possible to independently evaluate the killing of SIINFEKL-pulsed EL4 target cells by the same CAR T cells. Whether through OTI TCR- or CAR-mediated binding, stable conjugate formation with the target cells results in T cell degranulation and release of perforin, granzymes, inflammatory cytokines, and chemokines. **(B–D)** Brensocatib greatly inhibits the cytotoxicity of mouse CAR T cells but not human CAR T cells directed against EL4 target cells. Cytotoxicity of mouse CAR T cells generated from OTI TCR-transgenic T cells **(B,C)** directed against **(B)** EL4 cells loaded with SIINFEKL peptide or **(C)** non-peptide-pulsed EL4 cells expressing CD19. Target cell death was quantified across the range of effector: target (E/T) ratios shown as the percentage of specific release of preloaded cytoplasmic ^51^Cr from the target cells, relative to complete lysis with 1.0 M HCl (defined as 100%) and spontaneous ^51^Cr release in the absence of CD8^+^ lymphocytes (defined as 0%). **(D)** Cytotoxicity of human CAR T cells directed against EL4 cells expressing CD19. The values shown are the mean ± SEM across three independent experiments, with each data point in each experiment estimated in triplicate.

At this point, our data clearly suggested that DPP1 plays a far more prominent role in the processing of mouse granzymes from their zymogenic to active forms than for human granzymes. However, a potential caveat to this conclusion is that the target cells used up to this point were derived from different species and could have had different intrinsic susceptibilities to perforin/granzyme-mediated apoptosis. To further test whether the reduced target cell deaths genuinely reflected reductions in the amounts of proteolytically active granzymes released by the T cells, we tested mouse and human CD8^+^ T cells that ideally recognized the same target cell while also utilizing the same modes of antigen recognition and signal transduction. To devise such a model, we generated EL4 target cells that expressed either human or mouse CD19. Mouse and human CD8^+^ CAR T cells were then generated to express CARs that enabled T cell binding to the mouse or human CD19 antigens, respectively. By utilizing T cells from OTI transgenic mice as the source of the mouse CAR T cells, this model also enables us to evaluate the killing of SIINFEKL-pulsed EL4 target cells by the same T cells (Figure 3A). Consistent with the previous results (Figure 1), we found that killing of the SIINFEKL peptide-pulsed EL4 cells was strongly inhibited by brensocatib (Figure 3B). The killing of the (non-peptide pulsed) EL4 cells expressing mouse CD19 by the same OTI T cells expressing anti-CD19 CARs was inhibited to a similar degree when DPP1 was inhibited by brensocatib (Figure 3C). In marked contrast to the mouse CAR T cells, human CAR T cells remained just as potent in killing CD19-expressing EL4 cells, irrespective of exposure to brensocatib (Figure 3D). For each of the experiments depicted in Figures 3B–D, complexing the free Ca^2+^ by adding EGTA completely abolished cell death, once again confirming that such cell deaths were mediated entirely through the perforin/granzyme mechanism (data not shown). Four independent experiments in which the effector/target ratio was set to 2:1 showed that brensocatib exposure inhibited mouse CAR T cells by >90%, whereas killing by the human CAR T cells remained unaffected (Supplementary Figure S1A). Given that the same EL4 cells were the targets of both human and mouse CAR T cells, this major difference in sensitivity cannot be ascribed to differences in the cell death signaling pathways activated in the target cells. As before, DPP1 inhibition greatly reduced substrate turnovers for the mouse GzmA and GzmB but had minimal effects on human granzyme activities (Supplementary Figure S1B and C).

**FIGURE 4:**
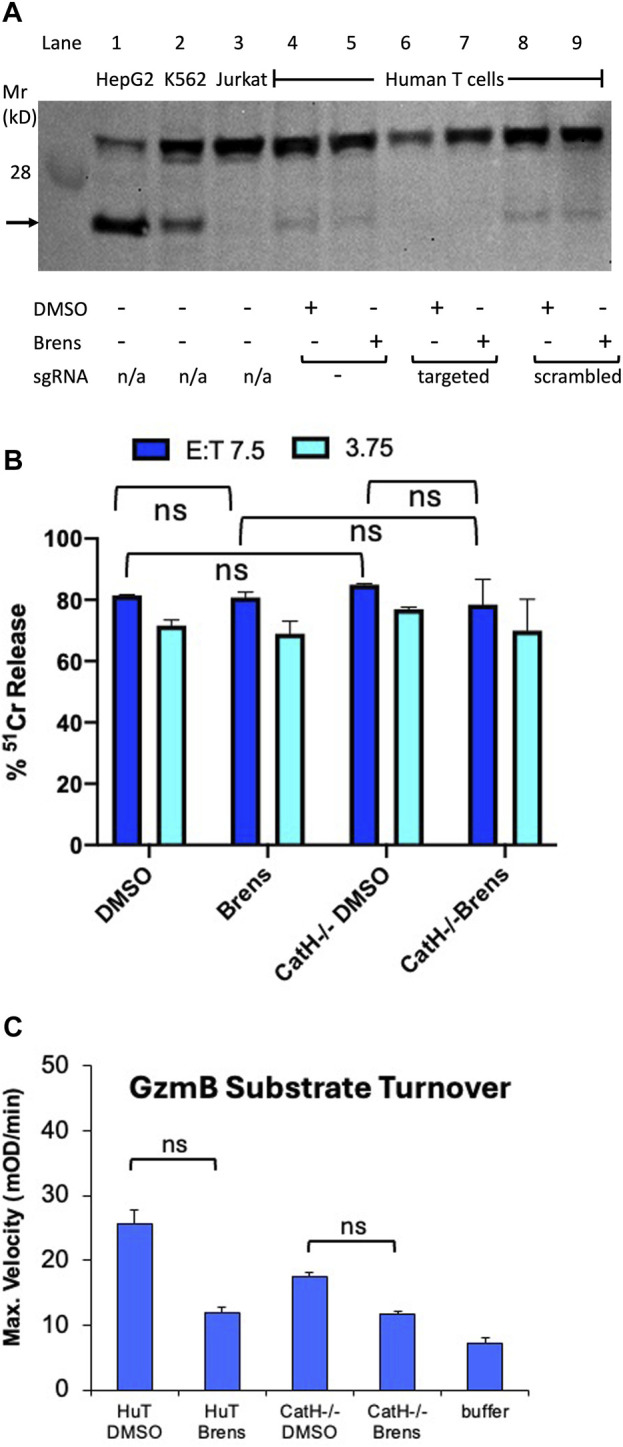
Loss of CatH expression by human T cells does not appreciably reduce their cytotoxicity. **(A)** Western blot of whole cell extracts of human activated CD8^+^ T cells expressing CRISPR-Cas9 along with either no oligonucleotide guides (lanes 4,5) or guides specific for *CATH* gene DNA (lanes 6,7) or guides with scrambled sequences (lanes 8,9). In lanes 1 to 3, extracts of HepG2 and K562 cells (which constitutively express CatH) and Jurkat cells (which do not) are shown as positive and negative controls, respectively. The blot was probed with an antiserum specific to the human CatH protein. The protein band corresponding to CatH is indicated by an arrow. Human primary T cells were cultured continuously in a medium containing 10 mM brensocatib or DMSO diluent. **(B)** IgG-mediated redirected killing of P815 target cells by activated human CD8^+^ CTLs at the E:T ratios indicated. The cytotoxicity was quantified as the percentage of specific release of preloaded cytoplasmic ^51^Cr from the target cells relative to complete lysis with 1.0 M HCl (100%) and spontaneous ^51^Cr release in the absence of CD8^+^ lymphocytes (0%). The CTLs were either wild-type for CatH expression or had the *CATH* gene disrupted by CRISPR-Cas9 as in **(A)**. The T cells were additionally cultured in a medium containing 10 mM brensocatib (Brens) or DMSO diluent. No killing was observed when anti-CD3 IgG was deleted from the cytotoxicity assay. **(C)** Lysates of the same T cell cultures as in **(B)** were assayed for turnover of the GzmB-specific tripeptide substrate. ns, not significant.

Given that brensocatib is a potent and specific inhibitor of human DPP1 *in vitro* (Table 1), the inability of brensocatib to inhibit human granzyme processing indicates the existence of alternative human convertases. We previously showed in mice that although DPP1 is responsible for the majority of granzyme convertase activity, the CTLs of CatC−/− mice still had some residual granzyme activity and that this was sufficient to ensure that CTL-mediated target cell death was not completely abrogated (Sutton et al., 2007). By breeding mice that were deficient for both DPP1 and cathepsin H (CatH) expressions, we demonstrated that much of the residual granzyme convertase activity in the CatC−/− CTLs was due to CatH (D’Angelo et al., 2010). Biochemically, this seemed quite feasible as the aminopeptidase activity of CatH is potentially capable of removing the N terminal dipeptide from the zymogenic form one residue at a time, albeit less efficiently than the DPP1 dipeptidase that removes both simultaneously. Consequently, we hypothesized that CatH may also be responsible for the residual granzyme convertase activity observed in human CTLs exposed to brensocatib. To test this possibility, we disrupted both human *CATH* alleles in the human primary T cells using CRISPR/Cas9. By sequencing the PCR products generated from the disrupted cellular DNA with the sense and antisense primers at the extremities of exon 3, we found that the *CATH* alleles had been disrupted in >99.9% of the generated sequences (Supplementary Figure S2). Moreover, RT-PCR on the purified cellular RNA confirmed that no detectable CatH mRNA remained in the gene-disrupted T cells (data not shown). Finally, Western blotting confirmed the absence of the CatH protein in the gene-disrupted T cells. An immunoreactive species of approximately 24 kDa was detected in the lysates of both HepG2 and K562 cells, which were included as the positive controls, but was absent from the Jurkat cell lysates (negative control). A signal of similar molecular mass was detected in the lysates of activated human T cells regardless of the cells being cultured in the presence or absence of brensocatib (Figure 4A). The signal was lost in the T cells that expressed CRISPR-Cas9 and oligonucleotide guides targeted specifically to the *CATH* gene sequence but not from similar T cell cultures that expressed CRISPR-Cas9 without the oligonucleotide guides or guides with scrambled sequences (Figure 4A). By adding 10 µM of brensocatib during the entire 5-day period of T cell activation, the *CATH* gene-disrupted T cells were made functionally devoid of both DPP1 and CatH activities. Nonetheless, the cytotoxicity of the human T cells was not appreciably reduced (Figure 4B). Consistent with these observations, the human granzyme substrate turnovers diminished but were not totally lost (Figure 4C). Overall, the data indicate that pro-granzyme activation in the human CD8^+^ T cells is far less dependent on both DPP1 and CatH than the mouse granzymes.

## Discussion

Despite recognizing antigens through very diverse mechanisms, the CTL, NK, LAK, and CAR T cells (collectively referred to as the cytotoxic lymphocytes) all achieve target cell deaths through one principal pathway, i.e., granule exocytosis. This mechanism involves the secretion of a cocktail of potent toxins stored in the secretory vesicles in their cytoplasm into the tight immune synapses formed with the cognate target cells (Voskoboinik et al., 2015). Given the intrinsic toxicity of the pore-forming protein perforin and the granzyme serine proteases with which it is packaged and released, a series of protective mechanisms have evolved to protect the killer cells from inadvertent damage owing to premature activation, mistrafficking, or leakage of the toxins into the T cell cytosols (Lopez et al., 2012; Stewart et al., 2012; Sutton et al., 2016; House et al., 2017). Granzymes acquire proteolytic activity only after reaching the secretory vesicles, where the lysosomal cysteine proteases remove an activation dipeptide from their amino terminus (McGuire et al., 1993). Even then, mature granzyme molecules remain inhibited by tight electrostatic binding with the proteoglycan serglycin and the acidic pH (typically 5.5–6.0) of that compartment (Sutton et al., 2016). Upon secretion into the immune synapse, the neutral extracellular pH and dissociation from serglycin finally allow full and unfettered granzyme potency. The most important protease for activating pro-granzyme zymogens is DPP1, which efficiently catalyzes the simultaneous removal of the two amino acids at the N terminus of the protein substrates (McGuire et al., 1993; Andoniou et al., 2007; Bird et al., 2009; House et al., 2017).

Unlike granzymes, DPP1 is expressed ubiquitously in the lysosomes of many cell types and plays crucial roles in activating serine proteases in the neutrophils, mast cells, and exocrine pancreas. The near-total reliance of serine proteases such as PR3, CatG, and NE on DPP1 to acquire activity has prompted the development of small-molecule DPP1 inhibitors to treat chronic purulent infections, such as those that complicate the management of pulmonary bronchiectasis (Korkmaz et al., 2010; Chalmers et al., 2017). Given that the pro-granzyme serine protease zymogens expressed exclusively in the CTLs and NK cells are processed through similar mechanisms, an important prerequisite for the use of DPP1 inhibitors in the clinic is that there be no impact on the antiviral activities of the CTL/NK cells. Our *in vitro* findings strongly predict that the likelihood of negative impacts on pro-granzyme processing/activation in cytotoxic lymphocytes will be low in human subjects in that the cytotoxicities of conventional CD8^+^ T cells, IL-2-activated NK cells, and gene-engineered CAR T cells against various target cells were minimally disrupted despite long-term continuous exposure to high concentrations of a reversible DPP1 inhibitor *in vitro*.

The mechanisms by which granzymes are activated from zymogens to active proteases have been studied extensively in mice but are hardly known for humans. Given that both the granzyme gene repertoire and functions of individual granzymes have been shaped by different species-specific evolutionary pressures in rodents and humans, it is important to test the DPP1 inhibitors in the human setting. In inbred mice, significant information has been derived from gene-engineered CatC−/− mice. The initial characterizations of these mice demonstrated their total dependence on DPP1 for processing serine proteases in myeloid cells as well as a marked reduction in granzyme activation that coincided with a loss of >80% of the CTL/NK cell cytotoxicity against the target cells *in vitro* (Pham, 2006). However, subsequent studies showed that despite this significant loss of granzyme activity, the CTL and NK cells of the CatC−/− mice were capable of imparting potent lethal hits resulting in target cell apoptosis, with similar kinetics to cell death as that induced by DPP1-sufficient killer cells (Sutton et al., 2007). *In vivo*, the DPP1-deficient NK cells and CTLs elicited during infection by the natural pathogen murine cytomegalovirus (MCMV) were indistinguishable from the wild-type cells but superior to those imparted by the CTL/NK cells of mice homozygous for null alleles of both GzmA and GzmB (GzmAB−/−) (Lopez et al., 2012). Collectively, these findings strongly suggest that while DPP1 is an important contributor to pro-granzyme processing in mice, additional convertases must exist. This issue was resolved when it was shown that the amino-exopeptidase CatH, which sequentially removes single amino acids from the N termini of substrate polypeptides, also contributes to pro-granzyme processing. The CTLs of mice null for the expressions of both DPP1 and CatH had significantly less granzyme activities than those of mice lacking DPP1 alone. Remarkably, however, a small amount of residual granzyme activity was detectable in the CatC−/− and CatH−/− CTLs, indicating that a minor third convertase must exist (D’Angelo et al., 2010).

The current study demonstrates that the molecular mechanisms of pro-granzyme activation differ significantly between mice and humans. There are many other ways in which human and mouse granzyme genetics, biology, and biochemistry could differ. While GzmA, GzmB, GzmK, and GzmM as well as the genes that encode these are common to both species, GzmH is unique to humans, whereas granzymes C, D, E, and F are unique to mice (Smyth et al., 1996; Bird et al., 2009). The mouse *GzmB* gene is also highly polymorphic in mice, with some naturally occurring alleles profoundly influencing host survival to important pathogens such as the MCMV (Thia and Trapani, 2007). Although it is clear that DPP1 plays an important role in activating pro-granzymes in both species, its contribution to granzyme activation in humans is much less prominent than in mice. Furthermore, while the mouse CatH is responsible for much of the granzyme convertase activity that is independent of DPP1, human CatH has only a minimal contribution. This is evident from the observation that CRISPR-Cas9-mediated disruption of the *CATH* gene does not appreciably reduce CTL-mediated cytotoxicity, even in the presence of the DPP1 inhibitor brensocatib (Figure 4). Although previous gene knockout studies in mice have estimated that only ∼5% of the pro-granzyme convertase activity is mediated by proteases other than DPP1 and CatH, the current study suggests that up to 50% of human GzmA and GzmB activities are generated independently of DPP1 or CatH. Further studies are needed to determine the identities of these additional convertases.

The results of *in vitro* studies described above are quite consistent with the phenotypes of human subjects born with total congenital deficiency of DPP1. Individuals with the rare autosomal recessive immunodeficiency disorder PLS present with marked deficiencies of neutrophil and monocyte/macrophage functions in childhood that manifest as recurrent infections of the gums (chronic gingivitis) severe enough to cause permanent loss of dentition in children as young as 6 years. To the best of our knowledge, deficiencies of CTL/NK functions have never been reported in any case of PLS; this includes any abnormal predisposition to any viral pathogen or any form of malignancy. Both these observations and the results of our studies allow us to conclude that brensocatib administration to human subjects is unlikely to result in any important reductions in CTL/NK cell activities.

Finally, our findings are important for drug development programs aimed at blocking CTL/NK-cell-mediated cytotoxic functions in the context of autoimmune or postinfectious immunopathologies in that inhibition of DPP1 convertase activity is clearly inadequate for this task. Given this finding, new drugs that inhibit the pore-forming activities of perforin (Welz et al., 2018; Spicer et al., 2022; Gonzales-Fierro et al., 2023) or the most potent cytotoxic granzyme GzmB (Turner and Hiroyaso, 2019; Zeglinski and Granville, 2020; Aubert et al., 2022) are more likely to achieve significant inhibition of T/NK cell cytotoxicity by direct interference with the key molecular components of the granular exocytosis pathway.

## Data Availability

The original contributions in the study are included in the article/Supplementary Material, further inquiries can be directed to the corresponding author.
