# Supplementary material for: Pharmacologic inhibition of dipeptidyl peptidase 1 (cathepsin C) does not block in vitro granzyme-mediated target cell killing by CD8 T or NK cells
Source: Front Pharmacol. 2024 Jul 3;15:1396710. doi: 10.3389/fphar.2024.1396710 (PMC11251990; doi:10.3389/fphar.2024.1396710)

FO2(DMSO Control- Run 228) versus F06(DMSO BG Crispr KO- Run 228)


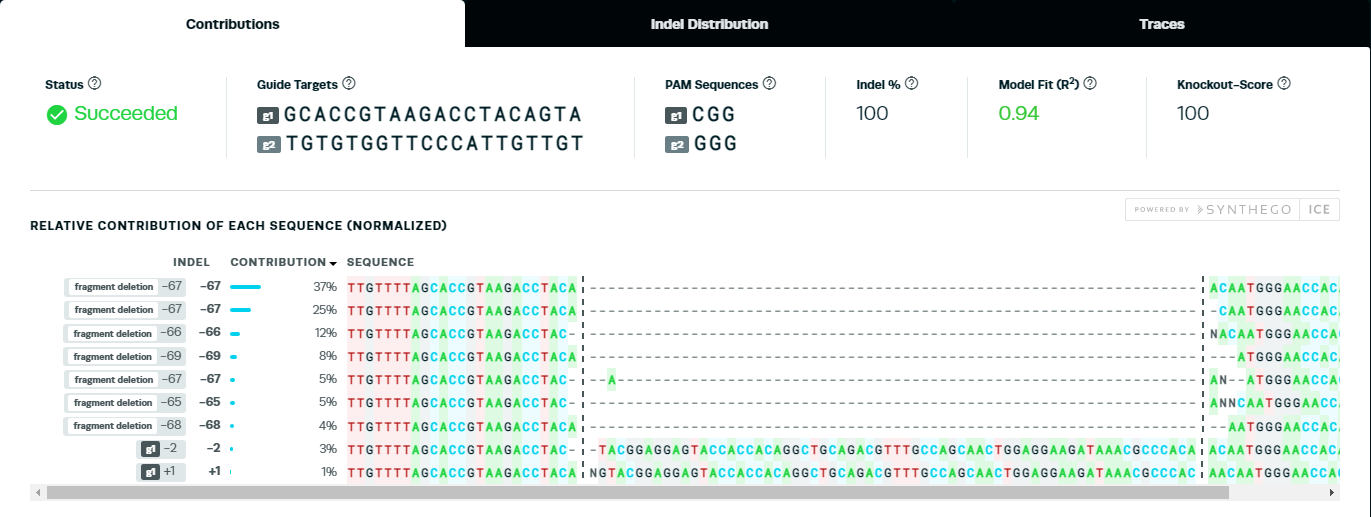

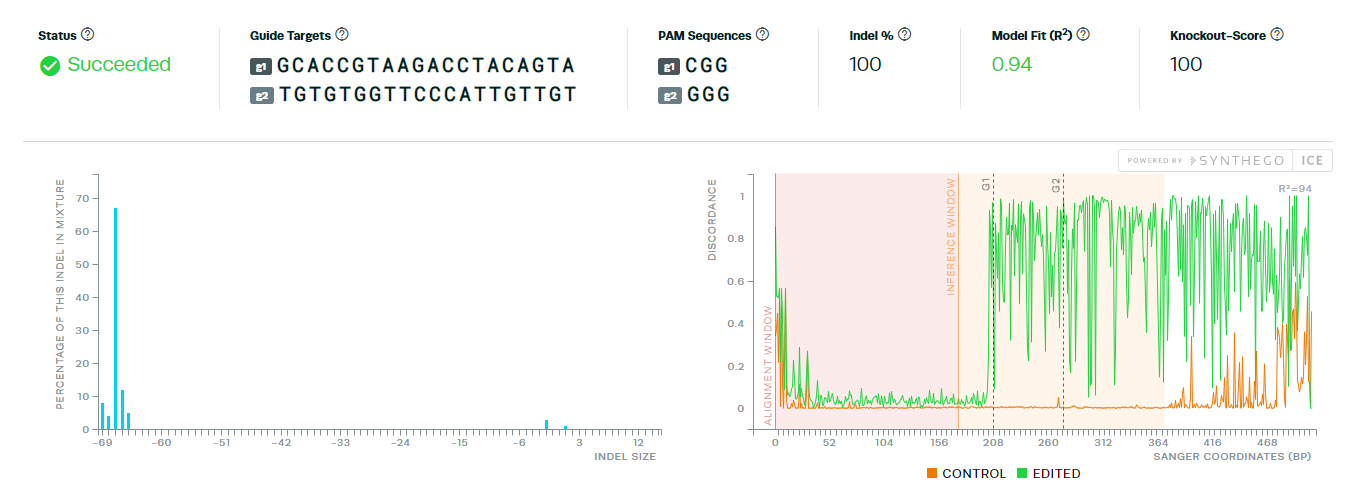

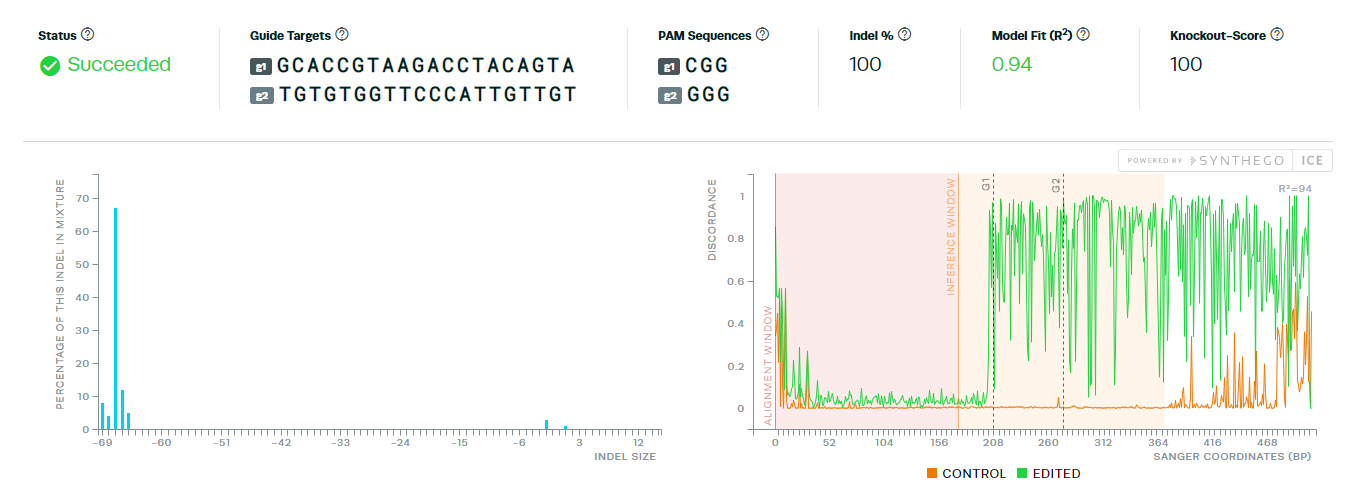

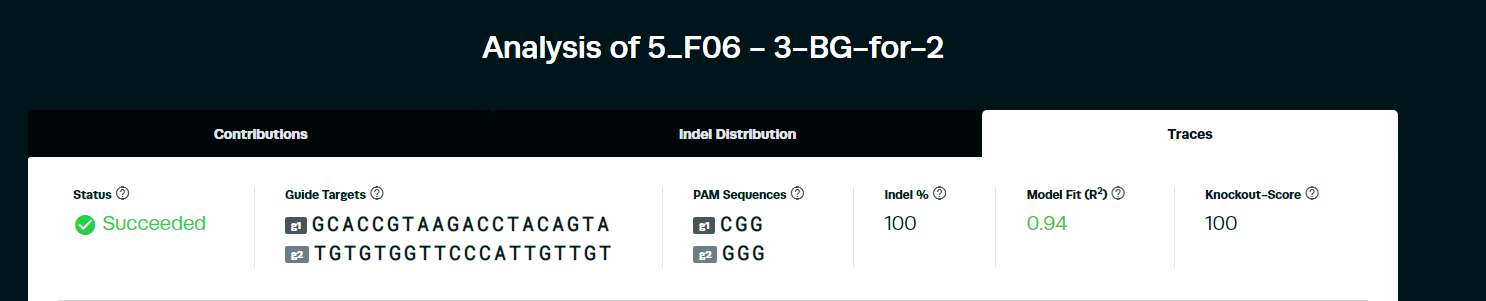


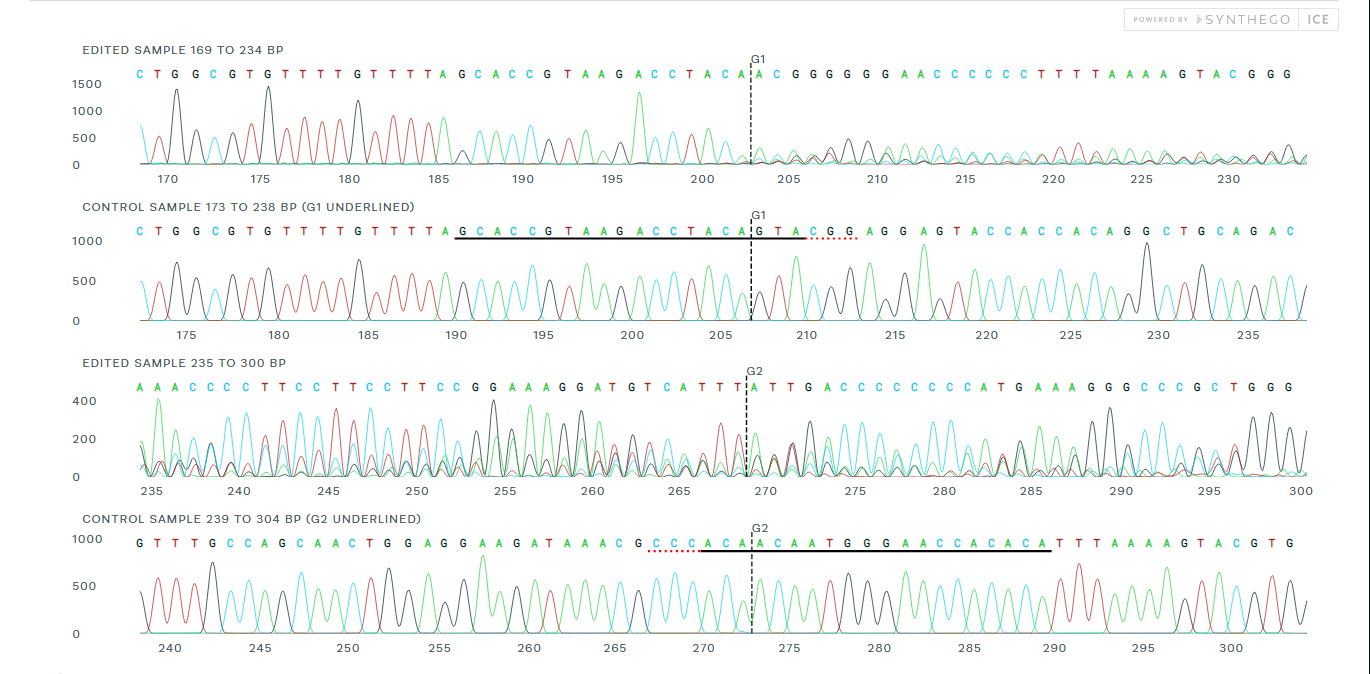


F04(Brenso Control- Run 228) versus H11(Brenso BG Crispr KO- Run 234)


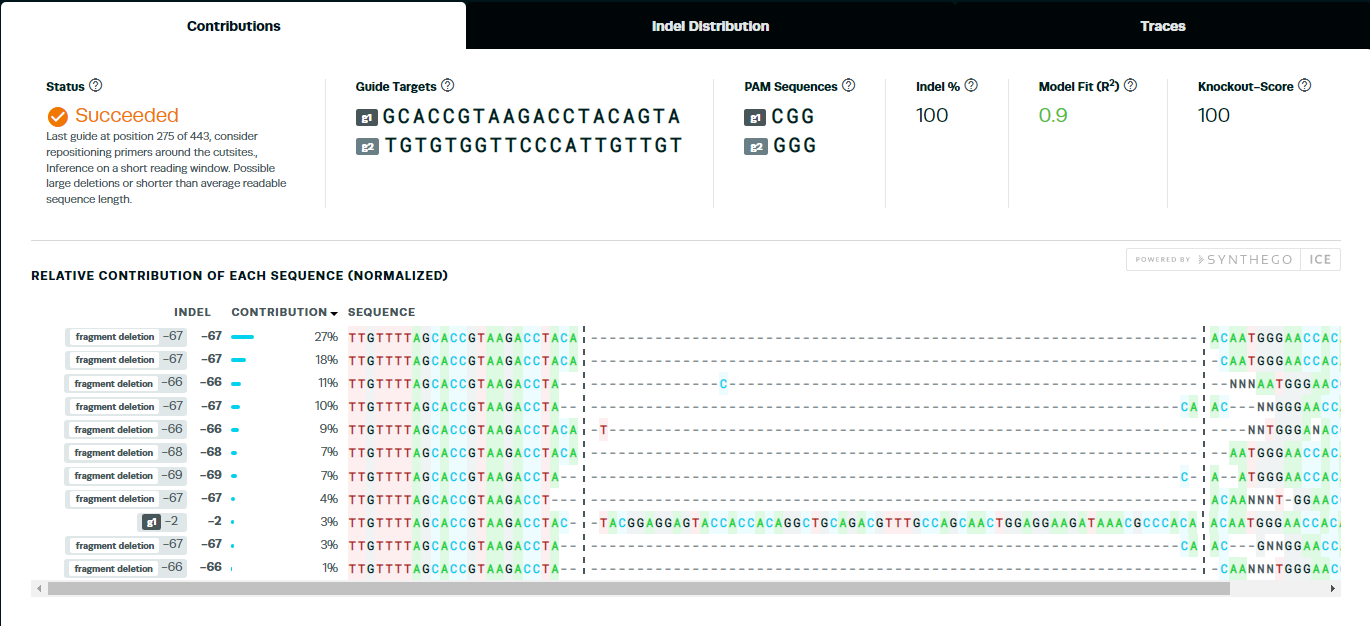


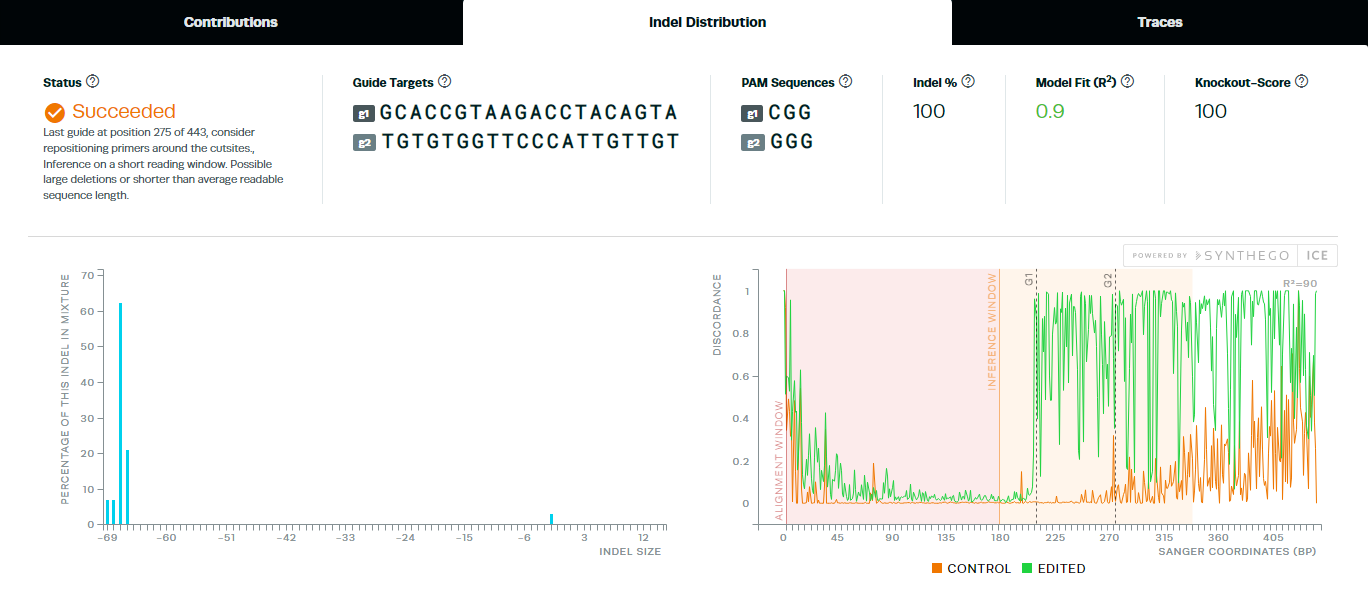

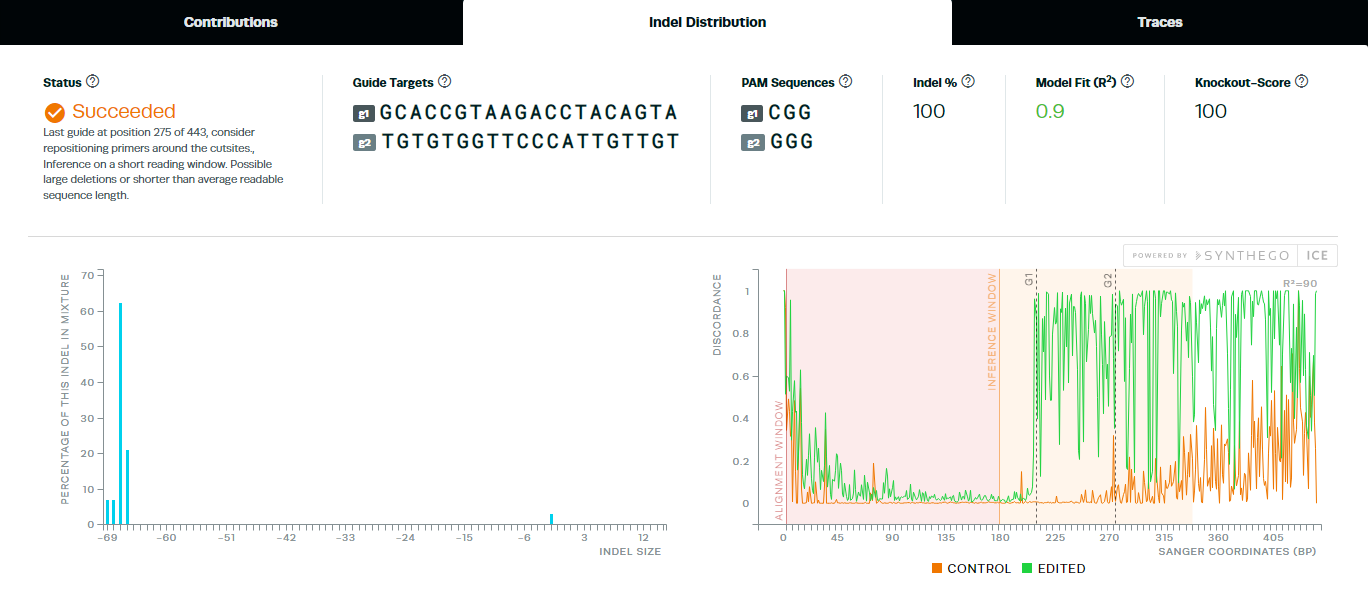


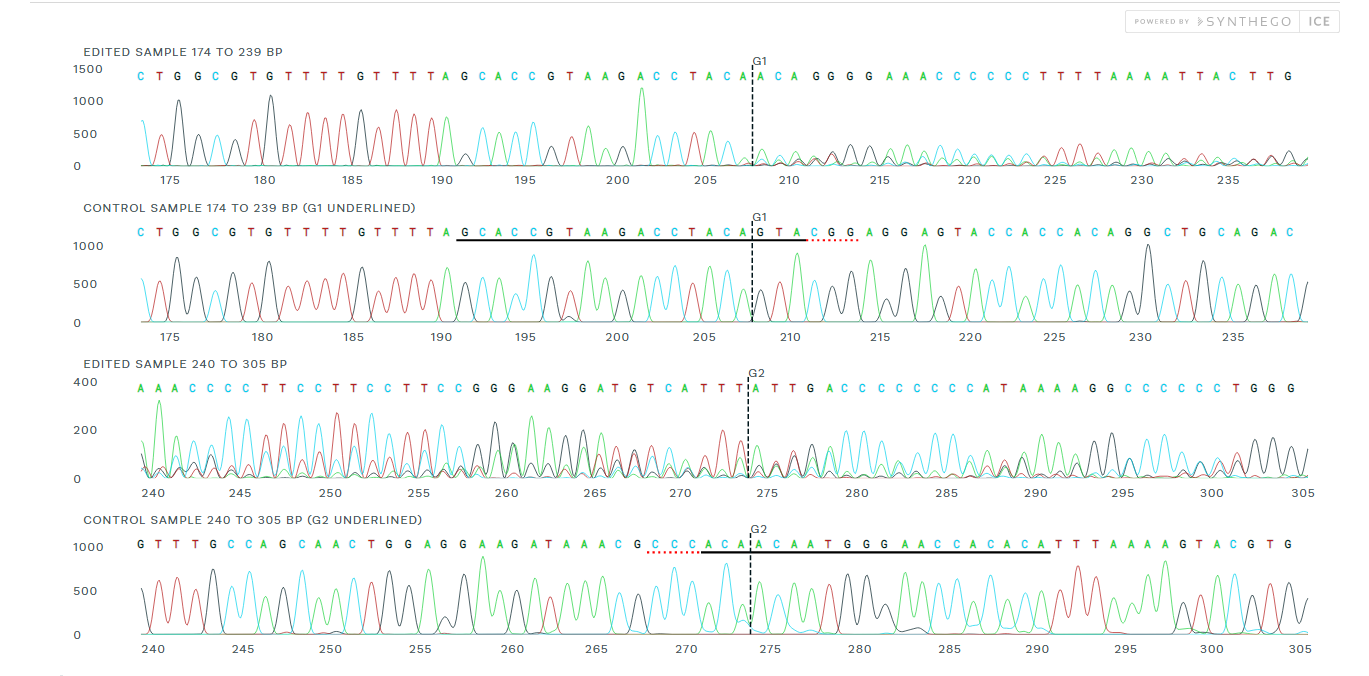


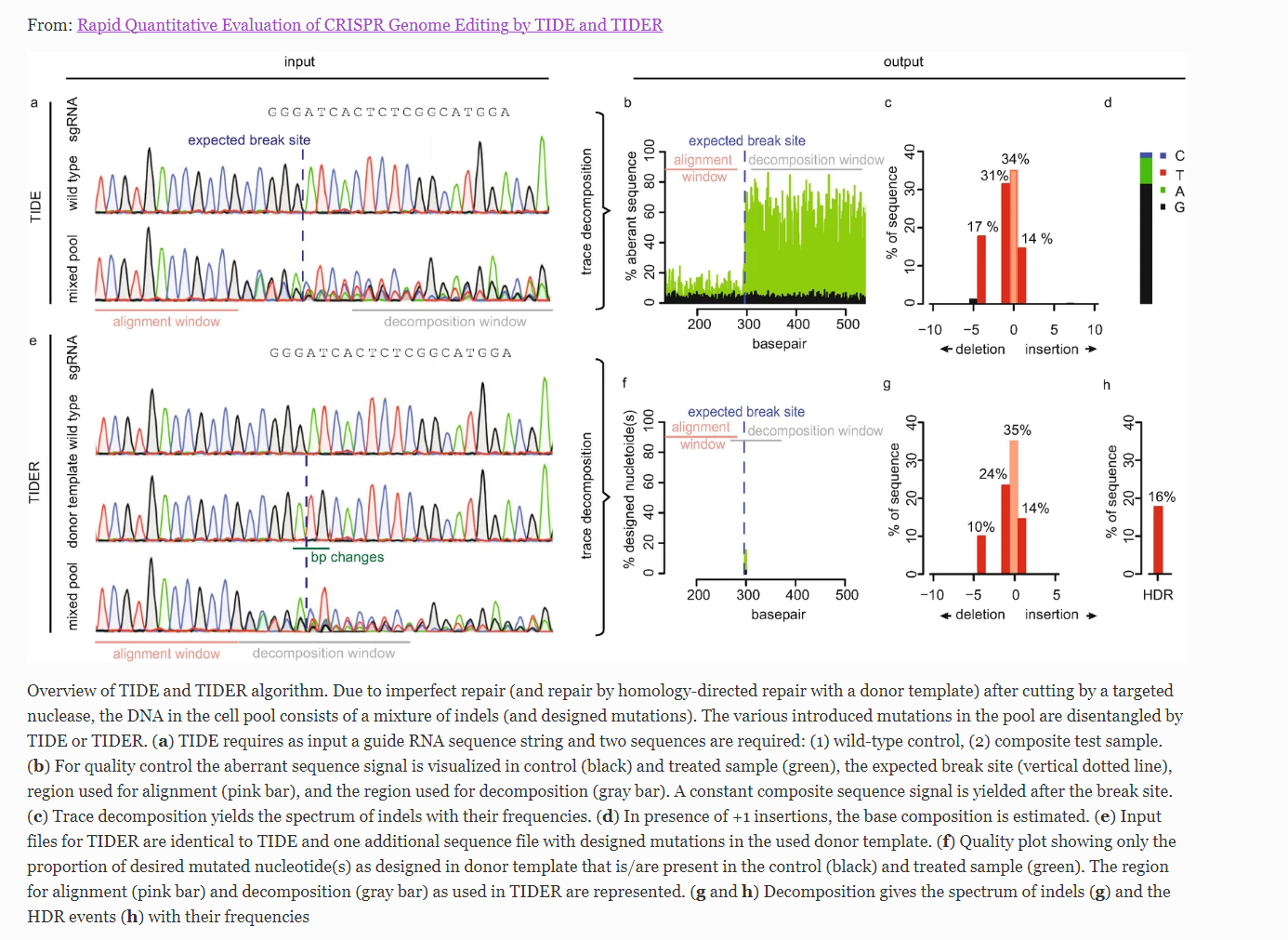

Supplement: Supplementary file 1 [file DataSheet2.zip › Fig data incl supp. Brens paper/Supp. Figure 2 /Supp Fig 2-Ice Ananlysis CatH Crispr KO.docx]
