# Supplementary material for: Pharmacologic inhibition of dipeptidyl peptidase 1 (cathepsin C) does not block in vitro granzyme-mediated target cell killing by CD8 T or NK cells
Source: Front Pharmacol. 2024 Jul 3;15:1396710. doi: 10.3389/fphar.2024.1396710 (PMC11251990; doi:10.3389/fphar.2024.1396710)
Supplement: Supplementary file 1 [file DataSheet2.zip › Fig data incl supp. Brens paper/Figure 4A/Cat H western 2nd Ab 170523.pptx]

## Slide 1
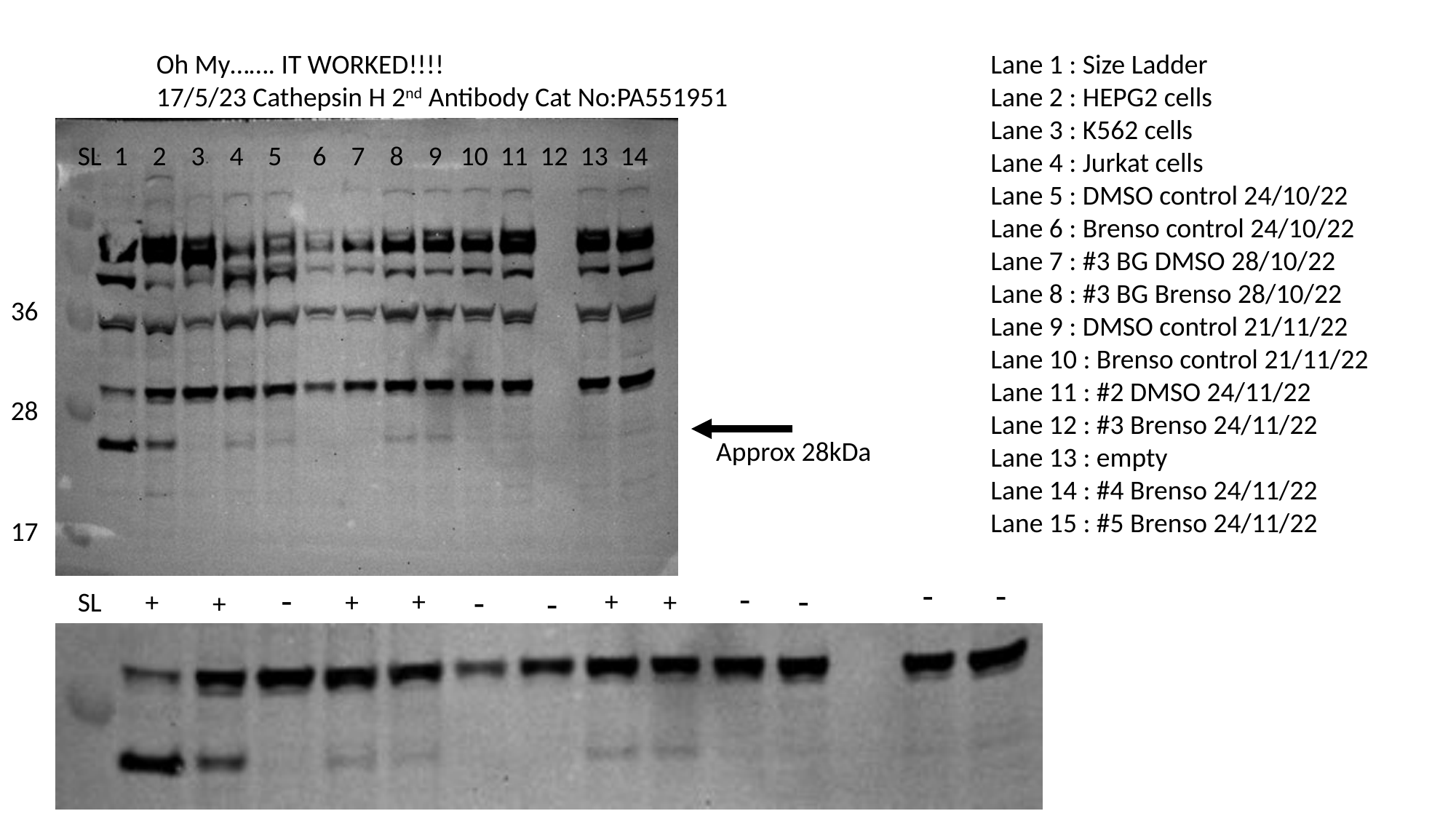

Oh My……. IT WORKED!!!!
17/5/23 Cathepsin H 2nd Antibody Cat No:PA551951
Lane 1 : Size Ladder
Lane 2 : HEPG2 cells
Lane 3 : K562 cells
Lane 4 : Jurkat cells
Lane 5 : DMSO control 24/10/22
Lane 6 : Brenso control 24/10/22
Lane 7 : #3 BG DMSO 28/10/22
Lane 8 : #3 BG Brenso 28/10/22
Lane 9 : DMSO control 21/11/22
Lane 10 : Brenso control 21/11/22
Lane 11 : #2 DMSO 24/11/22
Lane 12 : #3 Brenso 24/11/22
Lane 13 : empty
Lane 14 : #4 Brenso 24/11/22
Lane 15 : #5 Brenso 24/11/22
SL 1 2 3 4 5 6 7 8 9 10 11 12 13 14
36
28
Approx 28kDa
17
-
-
-
-
-
-
-
+
+
+
+
SL
+
+
