# Supplementary material for: Pharmacologic inhibition of dipeptidyl peptidase 1 (cathepsin C) does not block in vitro granzyme-mediated target cell killing by CD8 T or NK cells
Source: Front Pharmacol. 2024 Jul 3;15:1396710. doi: 10.3389/fphar.2024.1396710 (PMC11251990; doi:10.3389/fphar.2024.1396710)
Supplement: Supplementary file 1 [file DataSheet2.zip › Fig data incl supp. Brens paper/Supp. Figure 1A, B, C/supp figs 1 and 2.docx]

**Supplementary FIG. 1**


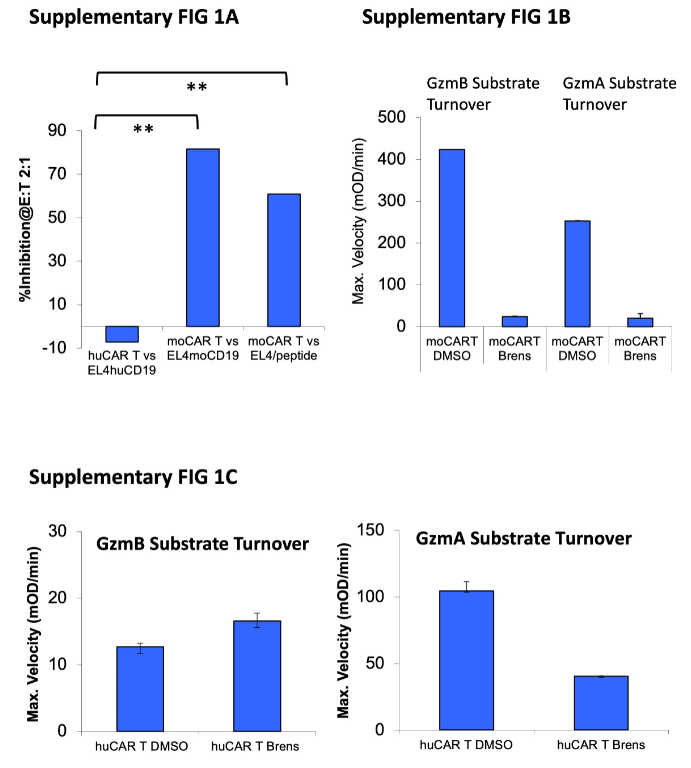


**Supplementary Figure 1.** **Brensocatib greatly inhibits the cytotoxicity of mouse CAR T cells, but not human CAR T cells directed against EL4 target cells. (A)** Cytotoxicity of human CAR T cells directed against EL4 target cells expressing CD19, or of mouse CAR T cells generated from OTI TCR-transgenic T cells directed against either EL4 cells loaded with SIINFEKL peptide or non-peptide-pulsed EL4 cells expressing CD19**.** Target cell death was quantified across four independent experiments at which the effector:target (E/T) ratio was set at 2. As in Figure 3 B-D, cytotoxicity was quantified as the percent specific release of pre-loaded cytoplasmic ^51^Cr from the target cells, relative to complete lysis with 1.0M HCl (defined as 100%) and spontaneous release ^51^Cr release in the absence of CD8+ lymphocytes (defined as 0%) (see Materials and Methods). **(B and C)** **Cleavage of tripeptide substrates specific for GzmB or GzmA in lysates of mouse CAR T cells (B) or human CAR T cells (C).** All cell cultures were in the continual presence of 10 µM brensocatib (Brens) or DMSO diluent. Values are shown as maximum reaction velocity and are the mean of 3 independent experiments (see Materials and Methods). ** p < 0.05 by unpaired t test.


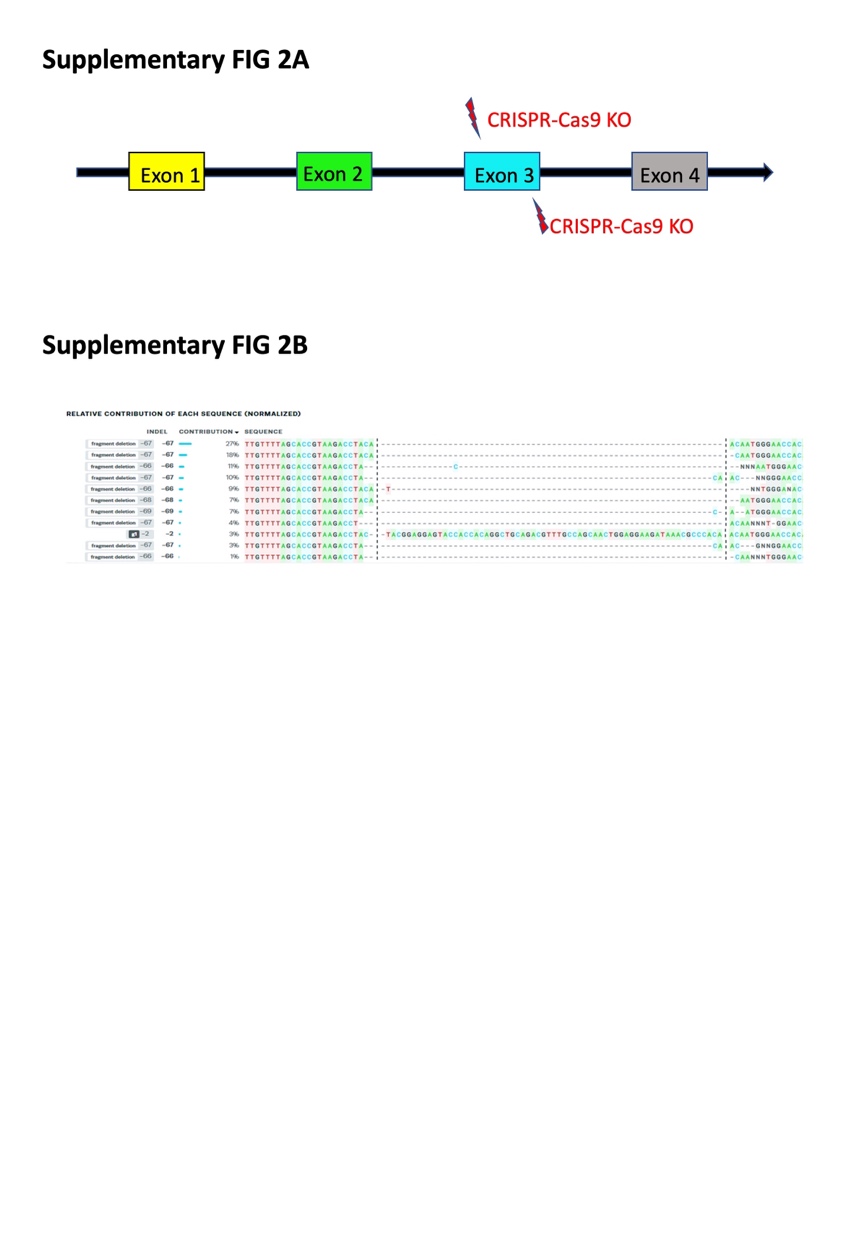


**Supplementary Figure 2. Evidence of effective disruption of the *CATH* gene by CRISPR-Cas9. (A)** Schematic representation of Intron/Exon organisation of the *CATH* gene showing approximate sites targeted by oligonucleotide guides 5’ GCACCGTAAGACCTACAGTA 3’ (sense) and 5’ TGTGTGGTTCCCATTGTTGT 3’ (antisense) and CRISPR-Cas9. **(B)** DNA sequences across the Exon 3 *CATH* site disrupted by CRISPR-Cas9 and the oligonucleotide guides in (A). The percentages shown indicate the contribution of that sequence to the total of all sequences detected (100%), to the nearest 1%.
