# Supplementary figures and images for: Pharmacologic inhibition of dipeptidyl peptidase 1 (cathepsin C) does not block in vitro granzyme-mediated target cell killing by CD8 T or NK cells
Source: Front Pharmacol. 2024 Jul 3;15:1396710. doi: 10.3389/fphar.2024.1396710 (PMC11251990; doi:10.3389/fphar.2024.1396710)

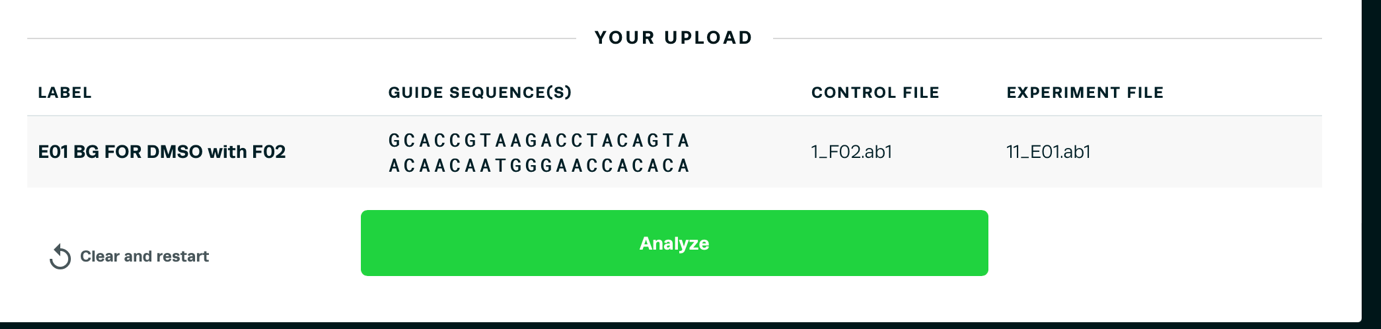

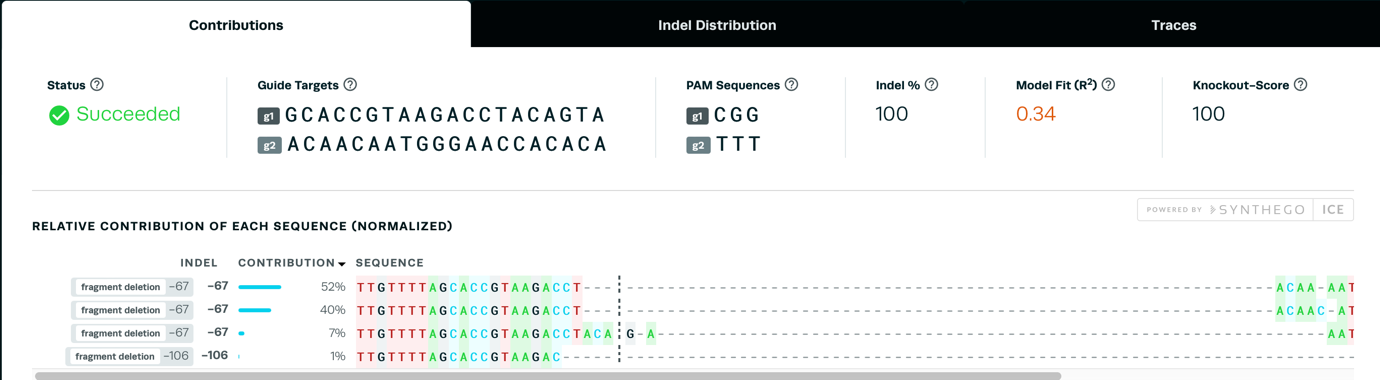

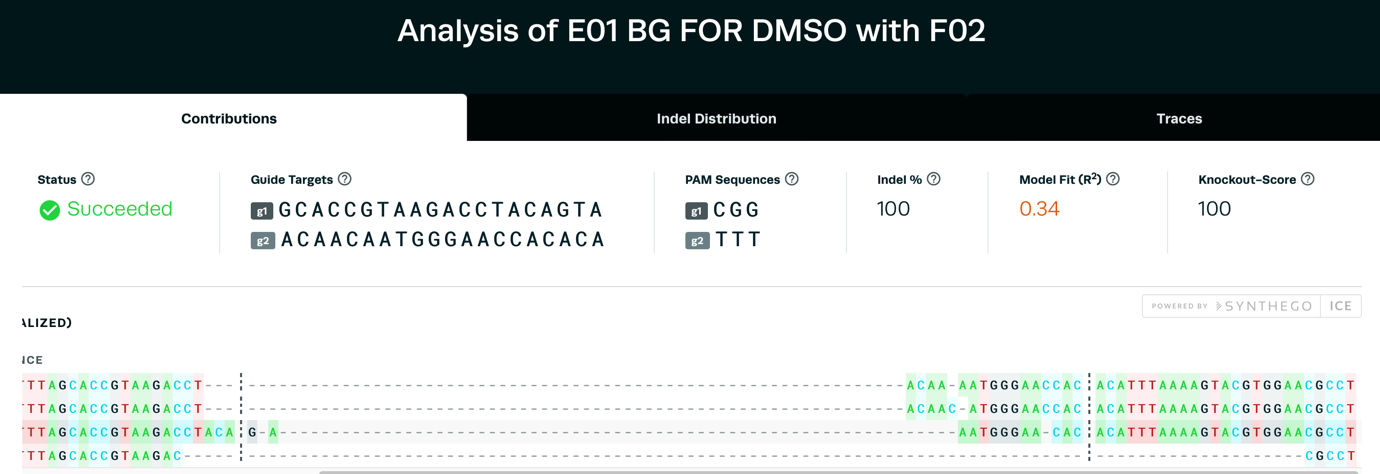

Supplement: Supplementary file 1 [file DataSheet2.zip › Fig data incl supp. Brens paper/Supp. Figure 2 /E01 For with F02.docx]

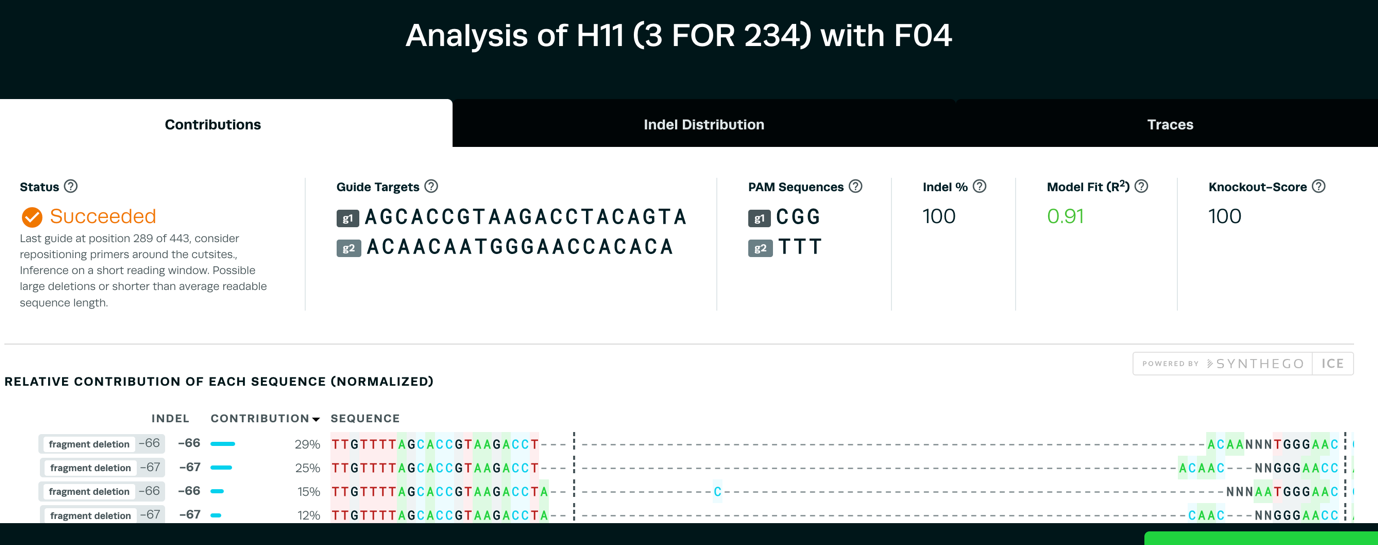

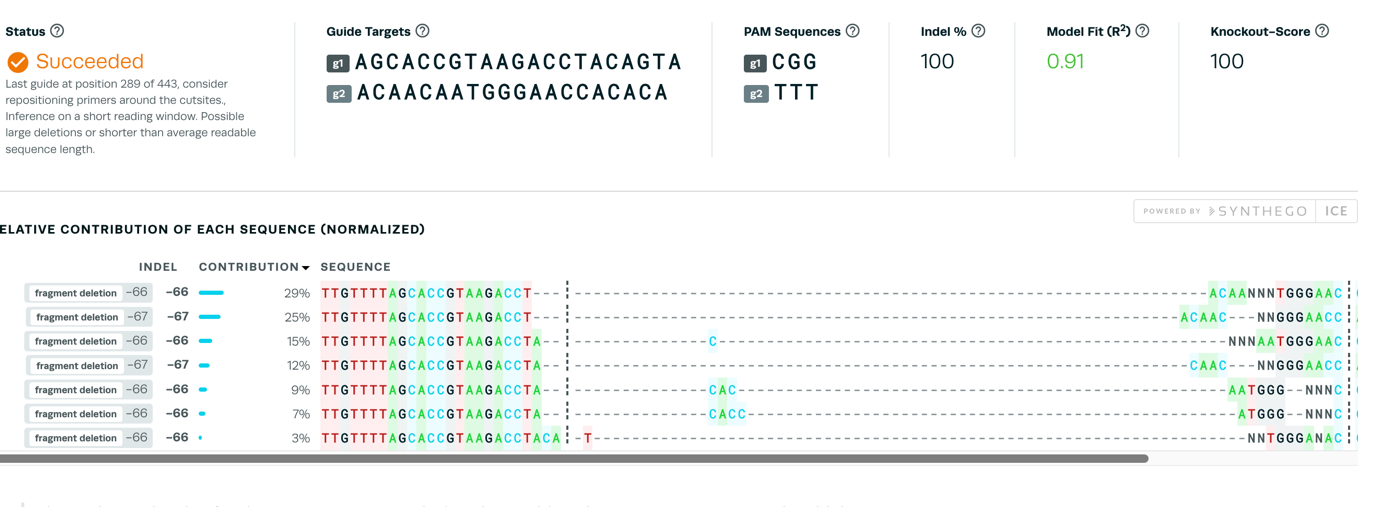

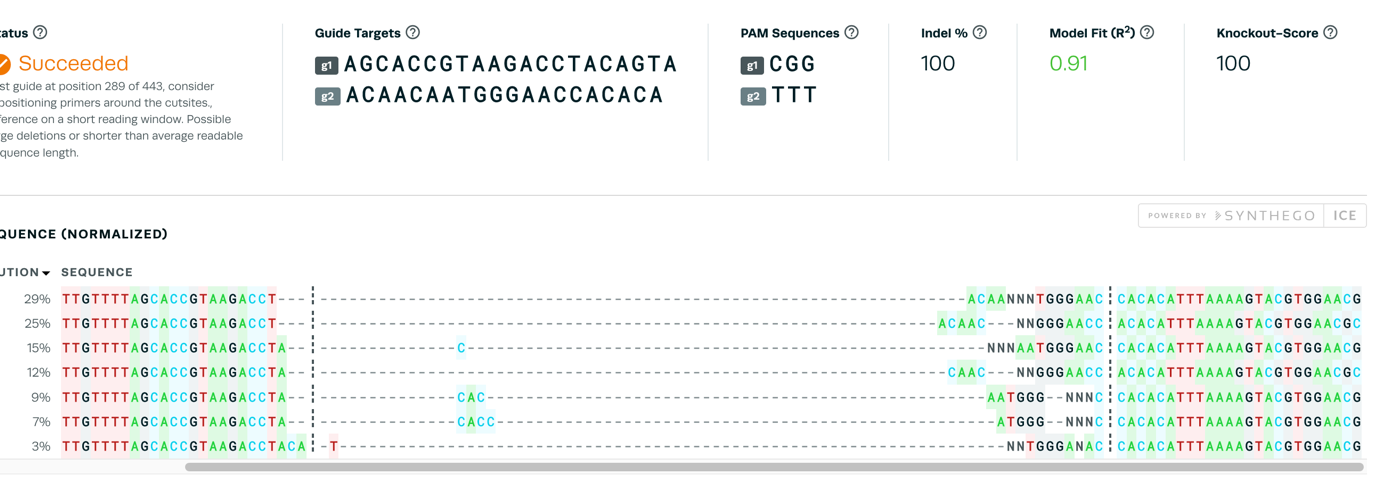

Supplement: Supplementary file 1 [file DataSheet2.zip › Fig data incl supp. Brens paper/Supp. Figure 2 /F04 (228 FOR) with H11 (#3).docx]

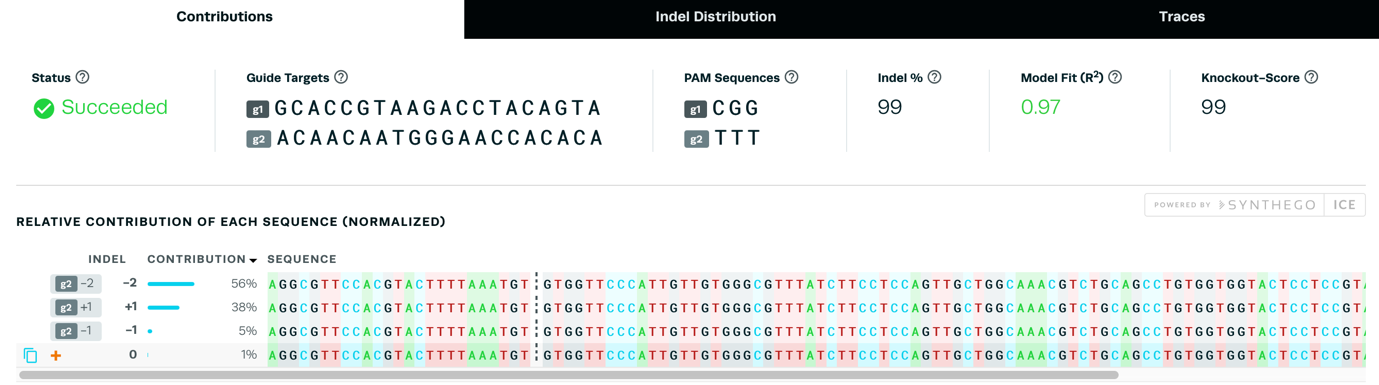

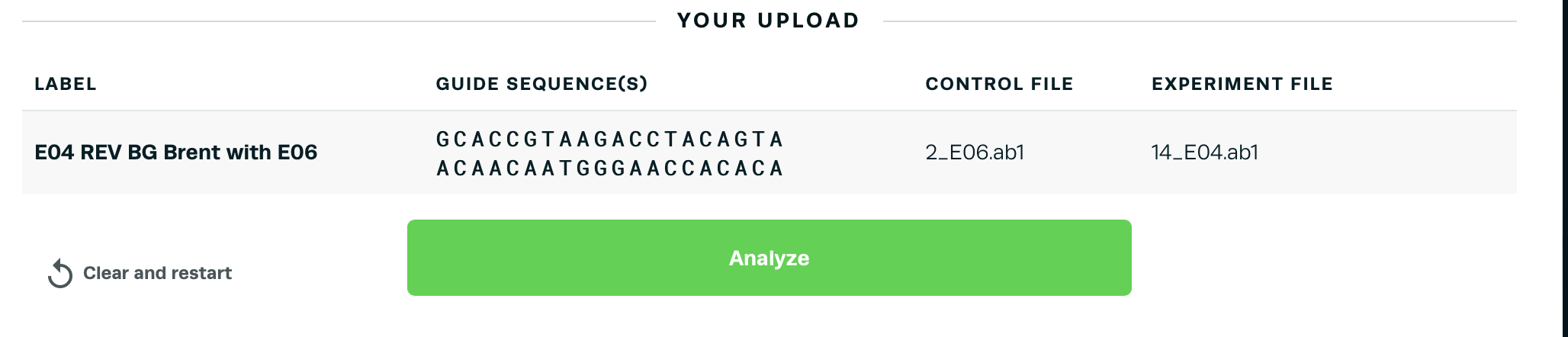

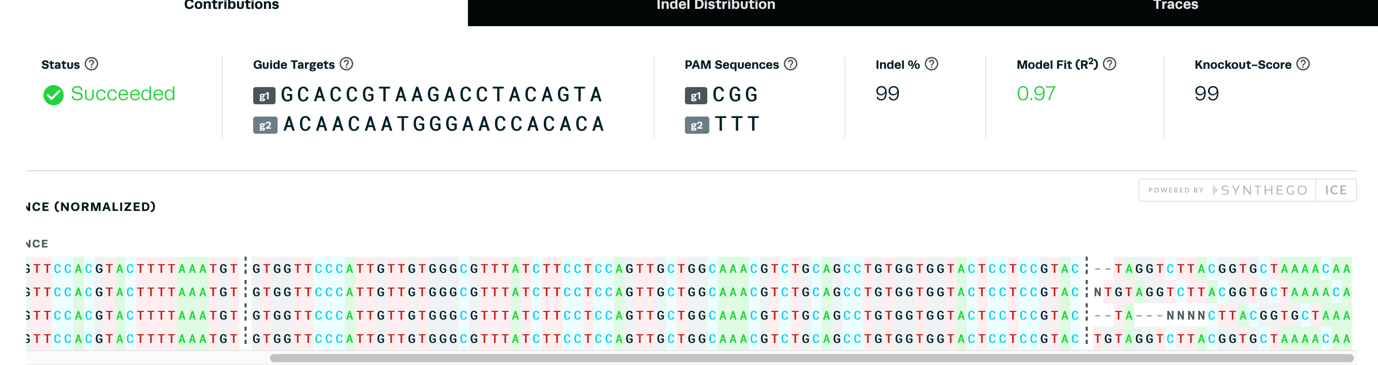

Supplement: Supplementary file 1 [file DataSheet2.zip › Fig data incl supp. Brens paper/Supp. Figure 2 /E04 BG REV with E06.docx]

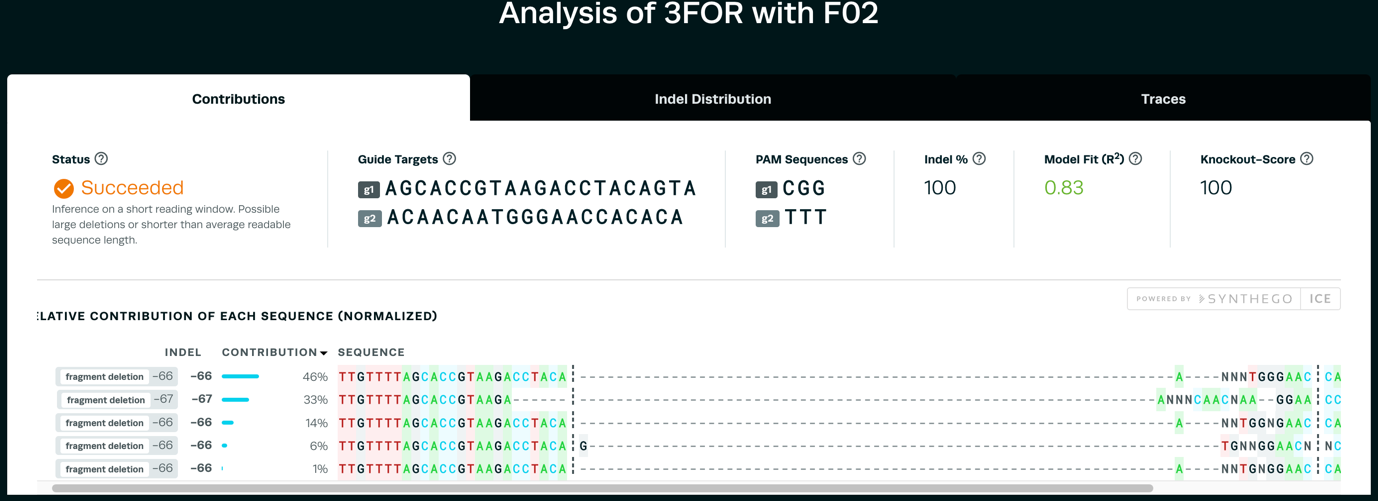

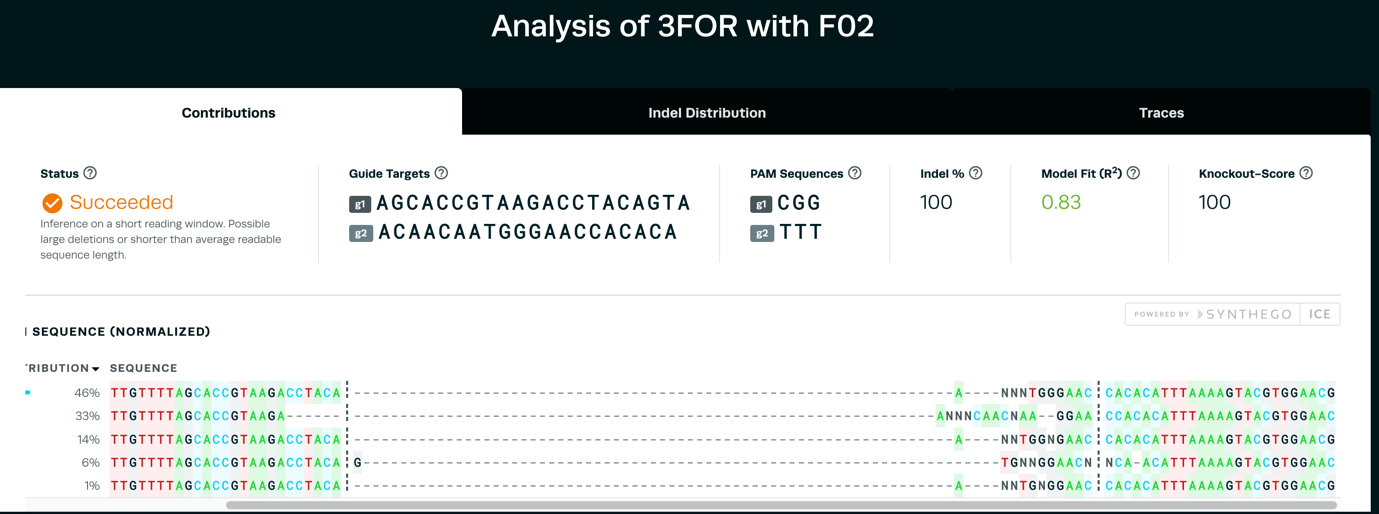

Supplement: Supplementary file 1 [file DataSheet2.zip › Fig data incl supp. Brens paper/Supp. Figure 2 /F02 FOR (228) with #3 FOR H11.docx]

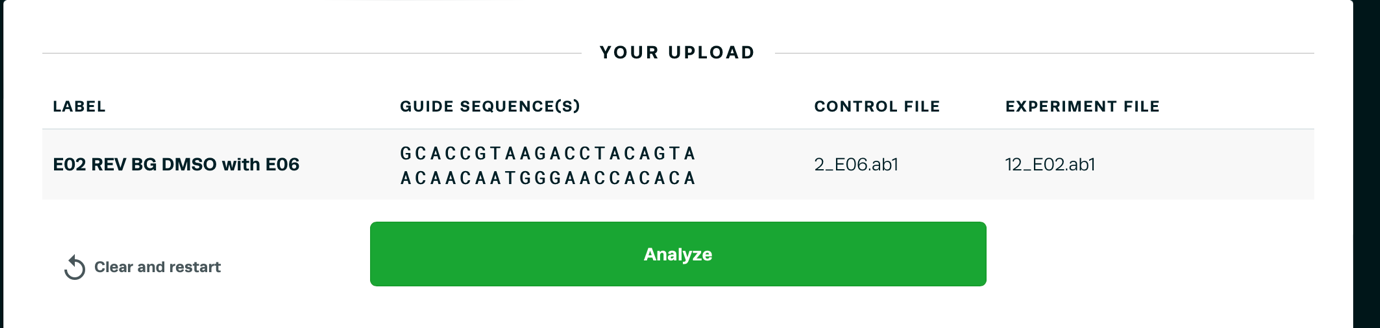

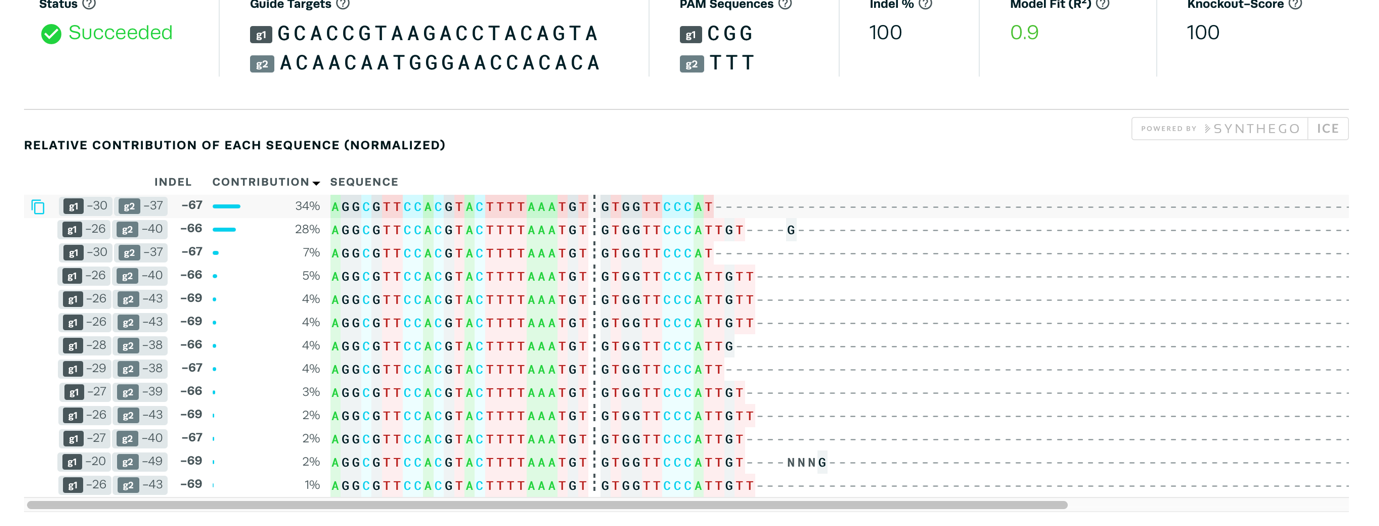

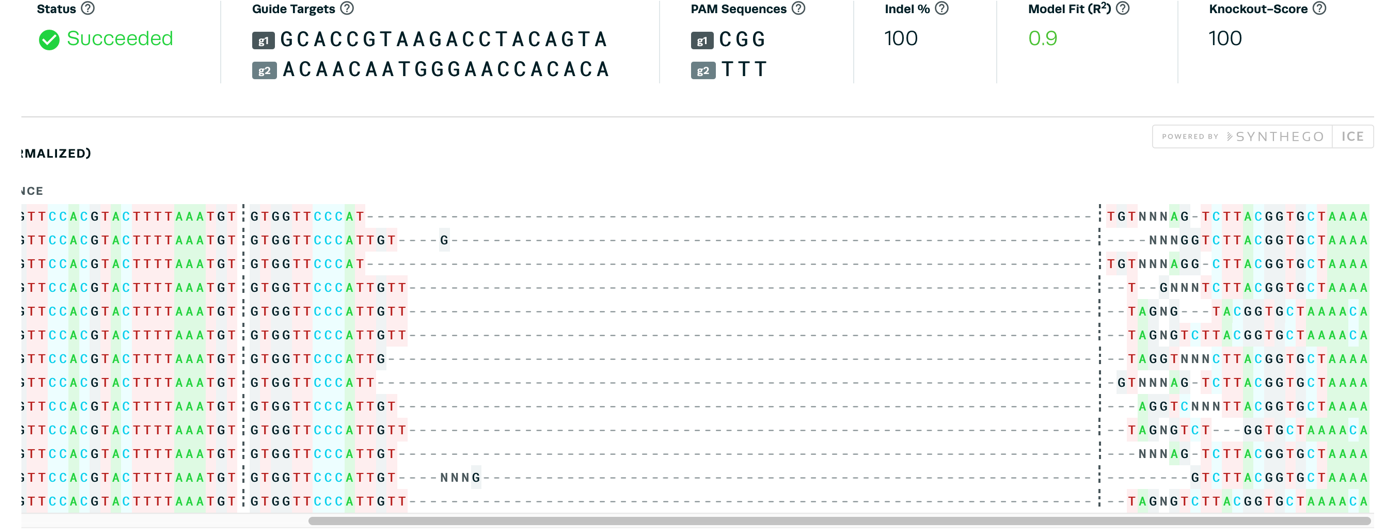

Supplement: Supplementary file 1 [file DataSheet2.zip › Fig data incl supp. Brens paper/Supp. Figure 2 /E02 BG REV with E06.docx]

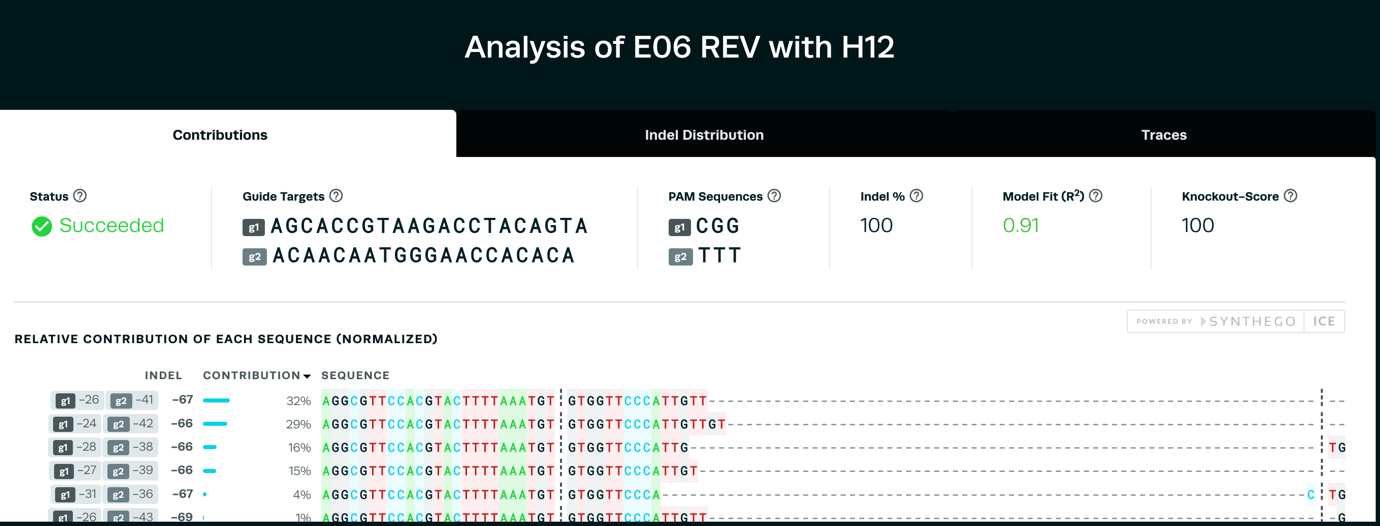

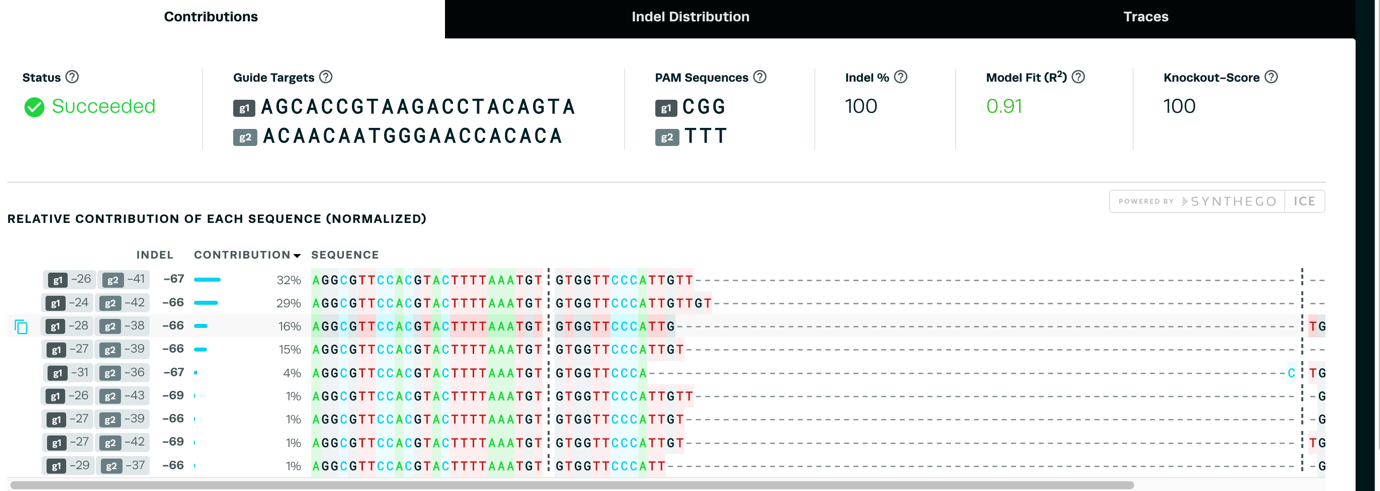

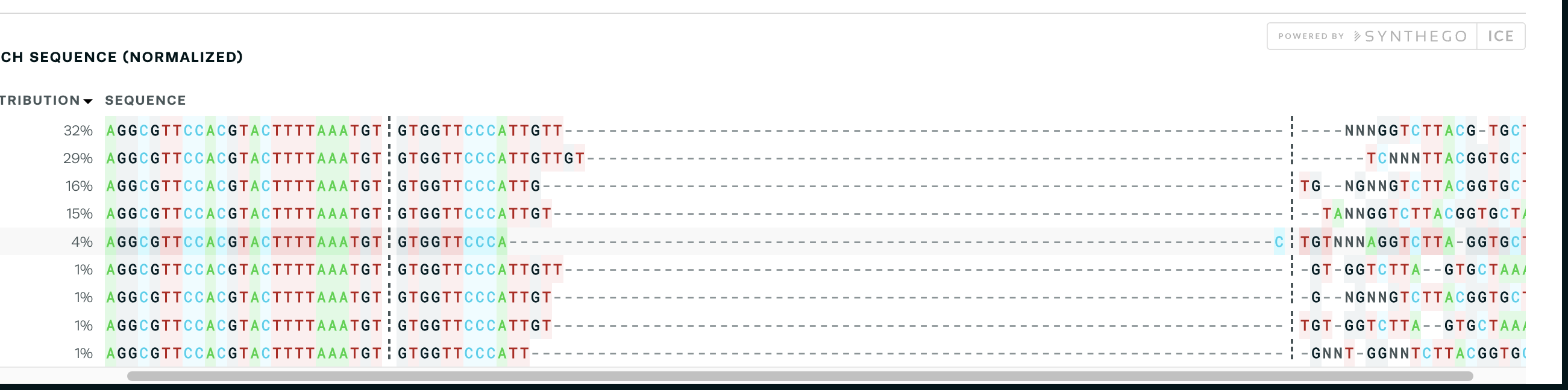

Supplement: Supplementary file 1 [file DataSheet2.zip › Fig data incl supp. Brens paper/Supp. Figure 2 /E06 REV with H12 (#3) .docx]

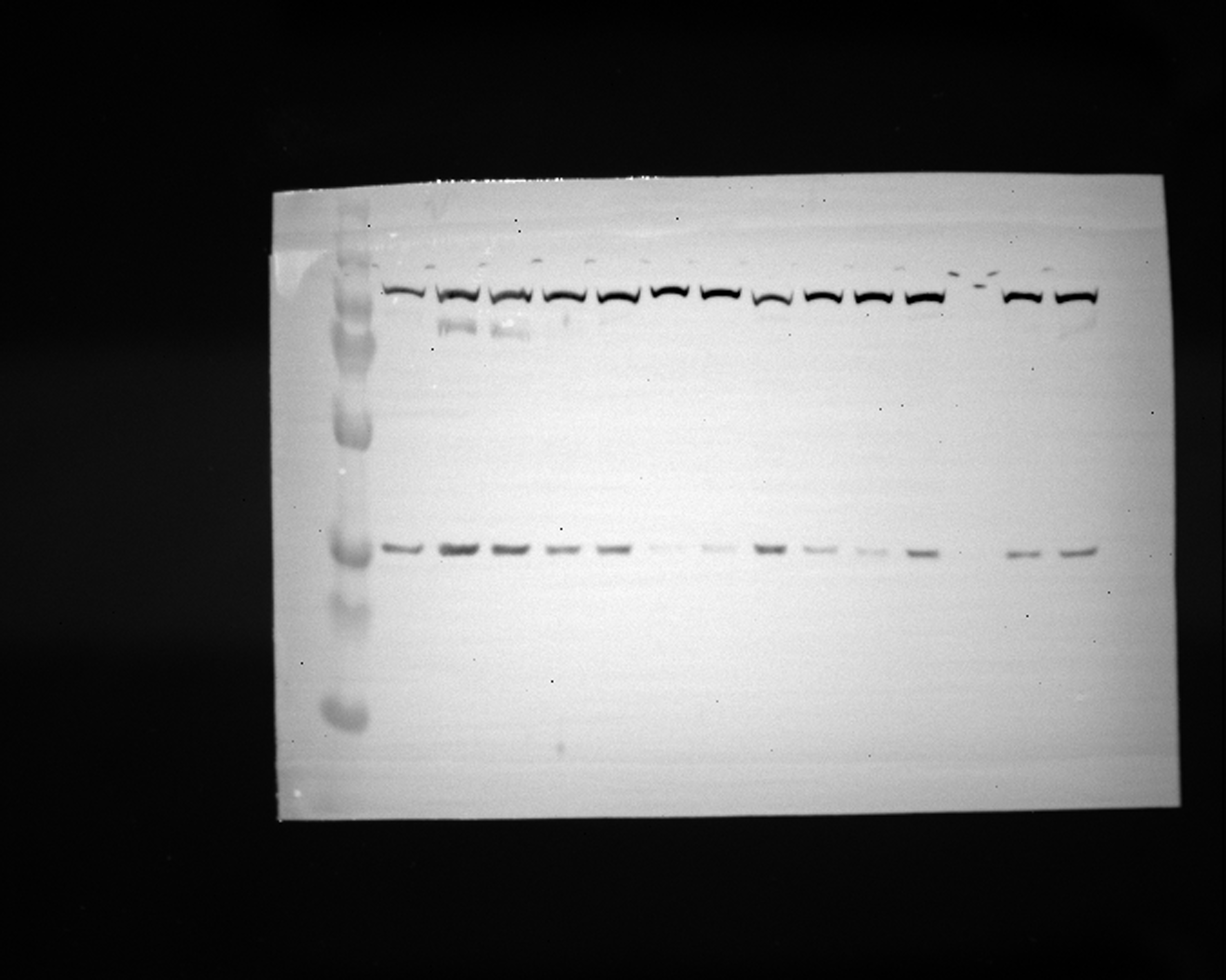

Supplement: Supplementary file 1 [file DataSheet2.zip › Fig data incl supp. Brens paper/Figure 4A/CHEMI_04052023_173453.tif]

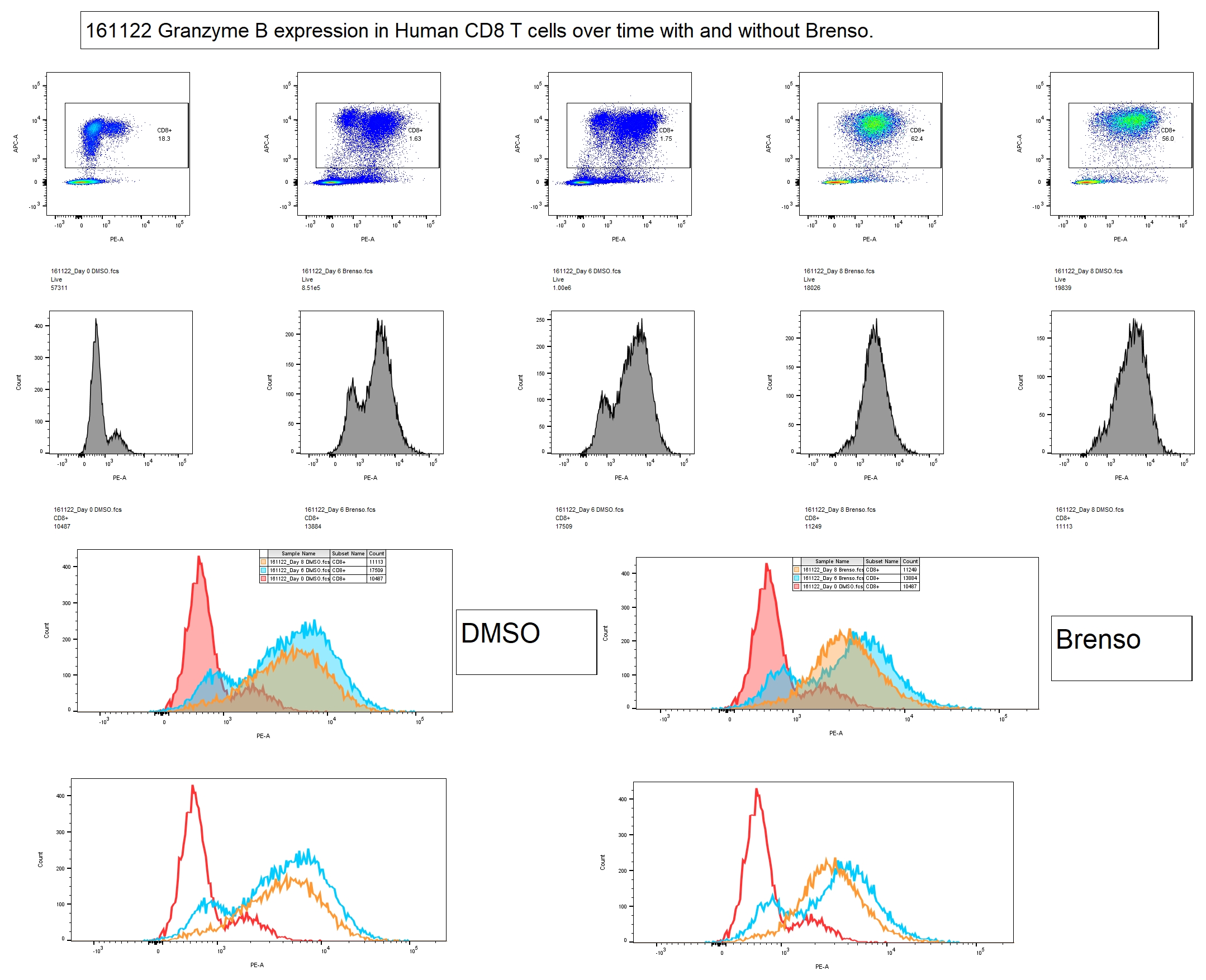

Supplement: Supplementary file 1 [file DataSheet2.zip › Fig data incl supp. Brens paper/Figure 2E/redo 080823-Layout.jpg]

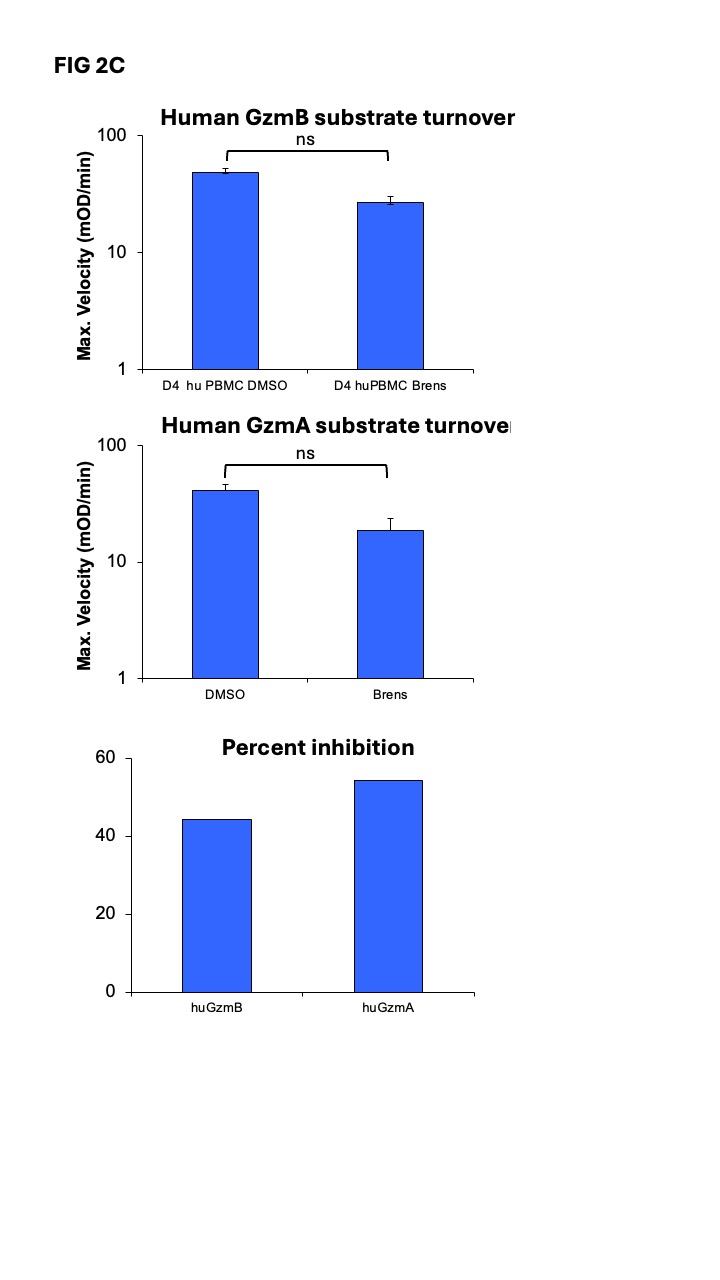

Supplement: Supplementary file 1 [file DataSheet2.zip › Fig data incl supp. Brens paper/Figure 2C huPBMC enzyme/Slide1.jpeg]

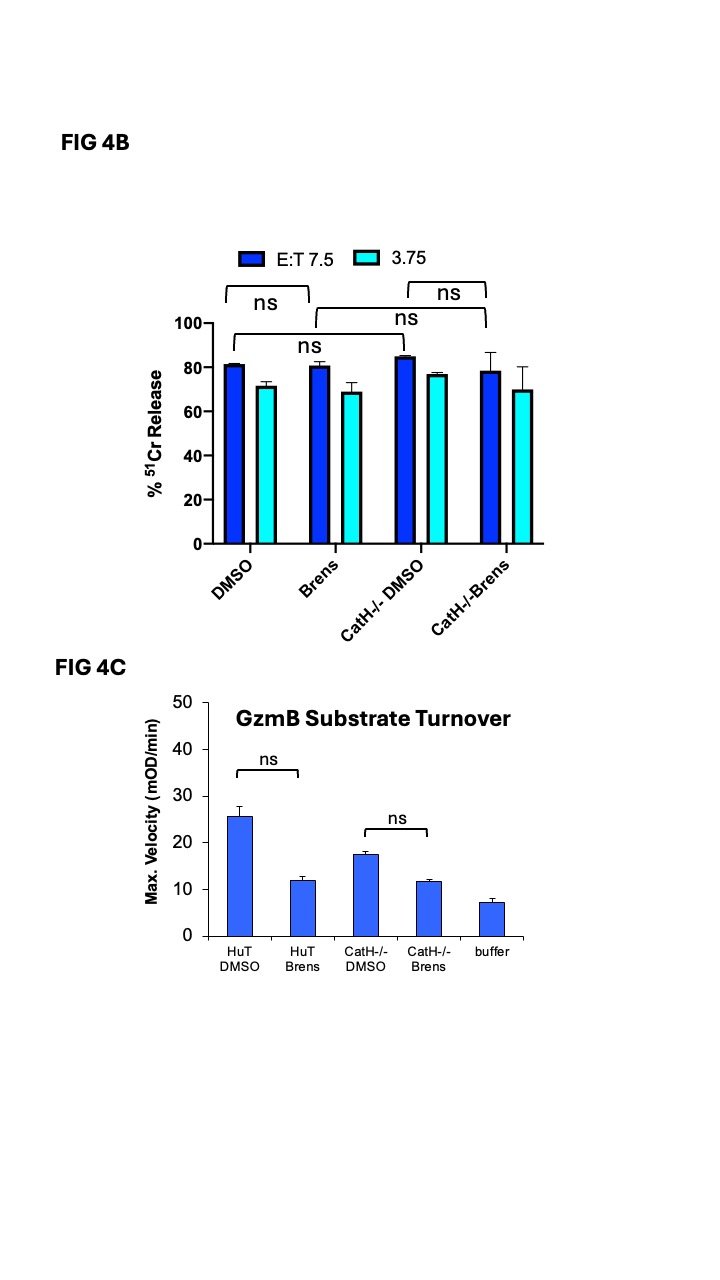

Supplement: Supplementary file 1 [file DataSheet2.zip › Fig data incl supp. Brens paper/Figure 4B/new 4B and C with stats/Slide1.jpeg]
